# A new method of task aggregation and optimization allocation for multiple groups collaborative task networks

**DOI:** 10.1038/s41598-025-11762-9

**Published:** 2025-07-29

**Authors:** WeiWei Du, XiaoWei Chen

**Affiliations:** 1 School of Mechatronics Engineering, Beijing Institute of Technology, Beijing, 100081 China; 2 North Automatic Control Technology Institute, Taiyuan, 030006 China; 3 State Key Laboratory of Explosion Science and Safety Protection, Beijing Institute of Technology, Beijing, 100081 China; 4 Advanced Research Institute of Multidisciplinary Sciences, Beijing Institute of Technology, Beijing, 100081 China

**Keywords:** Task networks, Multi-constraint multi-objective optimization, Task aggregation, Optimal task allocation, Adaptive genetic algorithm, Computational science, Engineering

## Abstract

As task diversity and inter-task relationship complexity grow, optimal formation power allocation is key to improving task execution efficiency. This paper proposes a task optimization allocation method with multiple groups collaboration, constructing a task network based on analysis of task static characteristics and inter-task relationship attributes. Firstly, the Lasswell 5W model is introduced to explore task characteristics, extend inter-task relationship types, and propose a generalized quantitative description method of the task network based on binary groups. Secondly, considering task allocation requirements, space–time constraints, resource capacity and demand constraints, the multi-group collaborative task allocation problem is transformed into a multi-constraint multi-objective optimization problem, establishing a multi-group task allocation mathematical model. Subsequently, the large-scale task network is decomposed into sub-task networks. The clustering cost function for tasks is constructed by analyzing the similarity between formation force locations and resource requirements, and between sub-task locations and resource requirements. The initial allocation strategy for subsets of formations and tasks is established. Finally, an adaptive mechanism is introduced to optimize the genetic algorithm’s crossover and mutation strategies, proposing a new adaptive optimal allocation algorithm for multiple groups tasks. Experimental results show that the proposed method achieves efficient task assignment under complex and diverse task planning scenarios.

## Introduction

### Motivation

Allocating the appropriate formation forces for each task is the key to ensuring efficient task execution and reducing execution risks. With the increasing complexity of the modernized battlefield environment and the dynamic changes in task requirements, the types of tasks present a strong diversity, and the complexity of inter-task relationships is also increasing. The problem of low model flexibility or poor adaptability exists when using specific assumptions or simplified models for multi-component task allocation, which is very likely to lead to mismatch of resources, delay of tasks, etc. Therefore, it is crucial to realize the adaptive optimal allocation of multi-group cooperative tasks by comprehensively considering factors such as task types, inter-task relationships and resource allocation.

At present, the challenges of adaptive optimal allocation of multi-group cooperative tasks are: (1) How to determine the task types and complex dependencies among tasks according to the attribute characteristics of the tasks, so as to lay the foundation for reasonable allocation of multi-group cooperative tasks; (2) How to dynamically optimize the allocation of resources for multiple groups on the basis of the limited resources, so as to solve the problem of the conflict between the actual resource demand and the existing limited resources; (3) How to achieve adaptive optimization of task allocation for multiple groups under the premise of meeting the task allocation requirements, time and space constraints, and so on. It can be seen that there is an urgent need to find an efficient intelligent task allocation method to solve the above challenges.

Task network is a kind of structured network that describes the interdependence and collaboration between multiple different tasks, which has obvious advantages in improving task allocation efficiency and realizing intelligent task management. In this paper, we try to construct a multi-group collaborative task network from the dimensions of task types, complex dependencies among tasks, spatial distribution of groups, and available resources, etc., so as to enhance the efficiency of inter-group collaboration by dynamically allocating a reasonable amount of tasks and resources to multiple groups.

### Related surveys and contributions

In recent years, multi-organizer task allocation has attracted extensive attention from research scholars at home and abroad, and the research focuses mainly on the formulation of allocation models and the design of algorithms^1^. With the development of intelligent algorithms such as deep learning and reinforcement learning, researchers and scholars have begun to use them to solve the problem of multi-organizer task allocation. For example, Peng et al.^2^ proposed a task allocation method based on multi-intelligent body architecture using deep reinforcement learning, which improved the allocation efficiency of large-scale tasks and concurrent tasks to a certain extent by building an automated mixed integer linear programming (MILP) framework; Aakriti Agrawal^3^ proposed a deep multi-intelligent body reinforcement learning method and used it to solve the problem of intelligent assignment of multi-robot tasks in dynamic logistics warehouses; Liu^4^ combined neural networks with graph theory and proposed a Graph neural network-Augmented Deep Reinforcement Learning scheme (GA-DRL), which established a Markov decision process model and shortened the task completion time; Li^5^ proposed an improved Multi-Agent Deep Deterministic Policy Gradient (MADDPG-D2) algorithm, which effectively improves the rationality of task allocation and reduces the complexity of decision making. These intelligent algorithms provide new research perspectives for multi-organizer task allocation, and excel in solving large-scale and dynamic allocation problems, as well as learning implicit laws. However, they also face some difficult challenges, such as high training data requirements, poor model interpretability, and difficulty in guaranteeing global optimization. This somewhat limits the development of intelligent algorithms such as deep learning for multi-organizer task allocation.

In fact, multicomponent task assignment is essentially a multiconstrained multi-objective optimization problem^6–8^. With the increase in the number of tasks, the limitations of multiple groups resources, etc., the allocation model will involve more constraints and decision variables. In this case, methods such as integer linear programming, bundling algorithms, and the Hungarian deterministic algorithm have difficulty in achieving high efficiency and good performance in task allocation. For example, Ye et al.^6^ proposed a decentralized task assignment method for the multi-task assignment problem with task coupling constraints by using a consensus bundling algorithm and verified the feasibility of the method in a search and rescue scenario. Bi et al.^9^ transformed the UAV formation power allocation into an integer linear programming problem and proposed a distributed multi-UAV task reassignment algorithm. This algorithm reduces the impact of UAV losses on the overall task execution effectiveness to a certain extent. Samiei et al.^10^ used an implicit coordination mechanism to propose a distributed matching algorithm based on the cloned Hungarian algorithm. This type of method is usually only applicable to multiple groups task allocation with deterministic tasks or fixed resource information, and fails to meet the dynamic task allocation requirements.

Compared with deterministic algorithms, heuristic algorithms, such as particle swarm optimization^11–13^, ant colony optimization^14–16^, and genetic algorithms^17–19^, show great advantages and potential in terms of task allocation efficiency and robustness. These heuristic algorithms have become a hot spot in the research of multiple groups task allocation. For example, Liu et al.^11^ established an effective and reasonable fitness function based on the consideration of the line-of-sight distance, relative angle, velocity, capture probability, and radiation source matching performance. Meanwhile, they proposed an adaptive simulated annealing particle swarm optimization algorithm. This algorithm basically realizes the interceptor attack task assignment. Zhou et al.^13^ utilized the Local Particle Swarm optimization to construct a decentralized target search model. This model improves the efficiency of coordinated UAV detection tasks in unknown environments. Wang et al.^14^ proposed a new solution construction method and pheromone updating rule based on the multi-objective ant colony system. This method achieves, to some extent, the enhancement of the system effectiveness. Ye et al.^18^ proposed a multi-objective optimization genetic algorithm for the NP combination optimization problem in multi-task scenarios. Ye et al.^19^ proposed a multi-machine task assignment method for construction base on improved genetic algorithms. This method lays a theoretical foundation for multi-robot collaboration in complex construction tasks. Bayrak et al.^20^ proposed a genetic algorithm with a custom coding, crossover, and fitness computation mechanism to assist in the development of airborne task assignment schemes. Mishra et al.^21^ analyzed anti-submarine warfare (ASW) task allocation and search path planning using the Hidden Markov Modeling Framework (AMMF). They proposed a two-phase method based on evolutionary algorithms, which solved the Non-deterministic Polynomial (NP) problem of polynomial complexity. This method was then used to solve the planning problem of coordinated tasks for anti-submarine warfare in dynamic environments. Ruan et al.^22^ established a task allocation model and a mathematical planning model for coordinated warfare under the constraints of temporal continuity to address the coordinated air combat task allocation problem. They solved the allocation problem by integrating linear programming algorithms, thus realizing the optimization of combat resources in the context of coordinated air combat. Kumar et al.^23^ proposed a hybrid model for distributed resource allocation by utilizing a hybrid genetic algorithm to address the issue of allocating server resources in a heterogeneous environment. This model reduces the response time of the communication system to a certain extent.

Among the above-mentioned heuristic algorithms, when compared with algorithms such as particle swarm optimization and ant colony optimization, the encoding of the genetic algorithm can more flexibly represent the attributes of tasks and resources. Meanwhile, the crossover and mutation operations help to prevent it from falling into local optimal solutions, which makes the genetic algorithm more suitable for handling the optimization problems of multiple types of tasks^24,25^. In addition, the robustness of the genetic algorithm enables it to cope with uncertainties and dynamic changes in task allocation, such as resource failures or changes in task priorities. In recent years, research scholars have proposed several advanced algorithms, including the Adaptive Genetic Algorithm (AGA)^26–28^, the Hybrid Genetic Algorithm (HGA)^29–31^, and the Hybrid Discrete Genetic Algorithm (HDGA)^32,33^. Among them, the AGA avoids premature convergence to a local optimal solution by dynamically adjusting the crossover probability and mutation probability. This enables it to efficiently identify the optimal solution for rational resource allocation during task allocation. Consequently, it addresses the issue of achieving optimal task resource matching, enhancing both resource utilization efficiency and task execution efficiency. The HGA integrates the genetic algorithm with other optimization algorithms, such as the simulated annealing algorithm and the particle swarm optimization algorithm. By combining the global search capabilities of the genetic algorithm with the local search capabilities of these other algorithms, the HGA enhances its convergence speed and accuracy. This allows it to solve task scheduling and resource allocation problems in complex task allocation scenarios, ensuring the scientific soundness and efficiency of the task allocation scheme. The HDGA is a derivative of the HGA algorithm. It is primarily employed to address discrete type task allocation problems, including discrete matching and task allocation within a discrete time window. The HDGA overcomes the issue of low search efficiency that traditional methods encounter in complex discrete spaces.

However, with the increase in the number of diverse tasks, the differences in attributes among different tasks, the complex relationships between tasks, resource competition mechanisms, and synergistic effects are prone to lead to the dual problems of insufficient coordination and inefficiency when genetic algorithms are applied. In addition, these methods fail to consider the balance of resource allocation during task allocation, which can easily result in resource waste. Especially when resources within the formation are limited, it will directly lead to low allocation efficiency. Therefore, it is necessary to study a new optimal allocation method based on the genetic algorithm that is applicable to diverse tasks and the complexity of their relationships.

Based on the above analysis, this paper constructs a multi-group task allocation network from the task attribute features of tasks and the relationships between tasks, and transforms the optimal allocation problem into a multi-constraint multi-objective optimization problem. This paper proposes an optimal multiple groups power allocation method based on an improved adaptive genetic algorithm, and illustrates the advantages of the proposed method in terms of optimal resource allocation and improving network execution efficiency by comparing it with AGA, HGA and HDGA algorithms.

The main contributions of this paper are as follows:

1. Aiming at the diversity of tasks and the complexity of inter-task relationships, this paper uses Lasswell’s 5W model to capture the multidimensional attributes of tasks. It extends the inter-task logical and functional relationships on the basis of the temporal and hierarchical relationships, and then proposes a binary-group-based generalized description of the task network. This description achieves a comprehensive characterization of the task attribute features and the inter-task relationships, and provides data support for the structured and systematic construction of the task network.
2. On the basis of comprehensively considering multidimensional constraints such as task time, space, and resource demand, the balance and efficiency of resource allocation are ensured by formulating a refined resource allocation strategy. Meanwhile, a mathematical model of task allocation for multiple groups group coordination is constructed. This model provides a theoretical framework and computational tools for solving the task allocation problem in a complex battlefield environment, and can address the issues of low task execution efficiency and resource waste caused by the complex decision-making process of task allocation.
3. A novel task hierarchical allocation model grounded in an adaptive genetic algorithm is proposed to tackle the issues of low efficiency and subpar quality when dealing with the solution of large-scale task networks. The model is capable of guiding the creation of high-quality initial solutions by leveraging the similarity among tasks. Additionally, it can dynamically adjust the optimization crossover strategy and mutation strategy to avoid the emergence of local optimal solutions, which is used to improve the adaptability and robustness of the algorithm to diverse tasks.
4. Combined with typical scenarios and test sets, the experimental results are analyzed qualitatively by compared with AGA, HGA and mixed HDGA methods. Through case studies, comparative experiments, and performance evaluations, the superior performance of the method proposed in this paper in enhancing task execution efficiency, optimizing resource allocation, and reducing execution risks is demonstrated.

The chapters of this paper are organized as follows. Section “A mathematical model of multiple groups for task allocation” introduces the mathematical model of multiple groups for task allocation. Section “Multi-component tasking modeling” presents a new adaptive genetic algorithm for task hierarchy allocation. Section “Experiment and analysis” analyzes and verifies the proposed method. Section “Conclusions” discusses the conclusions of this paper and future work.

## A mathematical model of multiple groups for task allocation

### Normalized description of a task network

To comprehensively and dynamically characterize task features and the relationships among tasks, and to enhance task allocation efficiency, this paper adopts a binary tuple description from two dimensions: task attribute features and inter-task relationship.

1 $$W = < T,R >$$

where $T = < T_{1} ,T_{2} ,T_{3} , \ldots ,T_{i} , \ldots ,T_{n} >$ is the task attribute feature set of n tasks, also known as the task list; *R* is the set of task relationships.

The Lasswell 5W model can comprehensively reflect the key characteristics of tasks in the domains of task planning such as emergency material transportation, intelligent management of lighting equipment, and the formulation of cultural communication plans, which helps to carry out structured and systematic analysis and description of the task, so as to optimize the execution and management of the task^34–36^. We map it into the task command theory, characterizes the task list from five aspects: What (task type), When (task time), Where (task space), Who (formations of execution), and Why (task objectives). What (task type) represents the task number, task name, and task requirements; When (task time) represents the start time and end time of the task; Where (task space) represents the area of operation of the task; Who (formations of execution) represents the location of the available formations and their capabilities; and Why (task objectives) represents the combat power requirements and the duration of the task.

Task attribute characterization can be expressed as:

2 $$T_{i} = < T_{type} ,\:T_{time} ,\:T_{space} ,\:T_{force} ,\:T_{index} >$$

where $T_{type}$ is the task type, expressed as $T_{type} = < p_{id} ,p_{name} ,CAP >$, $p_{id}$ is the task number, $p_{name}$ is the task name, $CAP$ is the task requires a vector of resources, such as electronic jamming equipment, detection equipment, transport vehicles, personnel, and other resources. $T_{time} = < t_{sta} ,t_{end} >$ is the task time, including the start of the task time $t_{sta}$, the end of the task time $t_{end}$. $T_{space} = < X,Y >$ is the task space, indicating the center of the combat task area, $T_{force} = < f_{Group} ,f_{sort} ,f_{scale} ,f_{dep} >$ is the implementation of the task of the combat group, group set $f_{Group}$, resource capacity $f_{sort}$, the number of groups $f_{scale}$, group location information $f_{dep}$ together to determine the use of combat resources, the implementation of the task of the efficiency as well as success rates $T_{index} = < I_{dam} ,I_{len} >$ is the task indicator, including the combat power requirement $I_{dam}$ and the task duration $I_{len}$.

The standardized description of task relationships is the fundamental premise for constructing task networks and organizing formations collaboration. Sequence relationship and hierarchy relationship, as important components of task relationships, provide a basic sequence and structural framework for task allocation, but are insufficient to cover all possible interactions and dependencies between tasks. In order to improve the feasibility and effectiveness of the allocation scheme, this paper expands the relationship between tasks on the basis of the sequence relationship and hierarchy relationship, proposing logical relationships and functional relationships to comprehensively characterize the connections between tasks. (1) Hierarchical relationships are represented as the connections between parent tasks and child tasks. (2) Temporal sequence relationships denote the sequential order of task execution. (3) Logical relationships signify the execution conditions among tasks, which are classified into three cases: “and”, “or”, and “not”. “and” indicates that the completion of the previous task is a prerequisite for the execution of the subsequent task. “or” implies that the two tasks are independent of each other. “not” represents a negation relationship, that is, another task is permitted to be executed only when a certain task has not been successfully completed. The description of logical relationships can effectively resolve conflicts between tasks and ensure the feasibility of the solutions. (4) Functional relationships refer to the impact of one task’s execution on the execution effects of other tasks. This paper categorizes them into facilitative relationships, obstructive relationships, and dependency relationships. The facilitative relationship means that the execution of the previous task has a positive impact on the subsequent task. The obstructive relationship means that the execution of the previous task has a negative impact on the subsequent task. The dependency relationship means that the execution of the subsequent task depends on the output information or action outcomes of the previous task. The introduction of functional relationships contributes to analyzing the synergistic effects and division of labor collaborations among tasks, as well as optimizing resource allocation and task scheduling.

Based on the above analysis, the relationship between tasks $T_{{\text{a}}}$ and $T_{b}$ can be described from four dimensions as Eq. (3).

3 $$R_{a - b} = < R_{level} ,R_{order} ,R_{\log ic} ,R_{fun} >$$

where $R_{level}$ represents the hierarchical relationship between $T_{a}$ and $T_{b}$; $R_{order}$ indicates the sequential relationship; $R_{\log ic}$ denotes the logical relationship; and $R_{fun}$ represents the functional relationship.

### Mathematical description of the problem

In order to intuitively understand the multiple groups task allocation problem, we describe the details considered in this study. Suppose there are n tasks in the task list that are collaboratively executed by m formations,$AG = \{ AG_{m} \} (m = 1,2, \ldots ,M)$. The attributes of the *m*th formation $AG_{m}$ are mainly considered from three aspects: (1) The average moving speed is $v_{m}$ km/min; (2) The position is $(X,Y)$ (unit: km, km). (3) The resource capacity vector is $GR_{m} = \{ GR_{ml} \}$, where $GR_{ml}$ is the l-th type of resource capability value possessed by $AG_{m}$. In fact, in the process of task execution, the resource capacity of the formation decreases with the increase of the number and intensity of the task. Therefore, after completing specific tasks, it is necessary to dynamically update the resource capacity of the formation.

4 $$GR_{ml}^{renewed} = GR_{ml} \left( {1 - \omega_{l} \frac{{GR_{ml}^{real} }}{{GR_{ml} }}} \right),l \in [1,L]$$

where $\omega_{l}$ is the loss coefficient of the $l$th type of resource capability, reflecting the degree of decay of the resource capacity over time or frequency of use, which is an attribute of the resource type itself, independent of the formation itself and the tasks performed, and usually takes a value in the range of 0 to 1, with 0 indicating no loss, and 1 indicating complete loss. $GR_{ml}$ is the value of the $l$th type of resource capability that $AG_{m}$ possesses before performing a task. $GR_{ml}^{real}$ is the actual consumption value of the $l$th type of resource capability during the execution of that task. $GR_{ml}^{renewed}$ indicates the resource capability value after the task is performed. The updated resource capability value is as Eq. (5).

5 $$GR_{m}^{renewed} = \{ GR_{m1}^{renewed} ,GR_{m2}^{renewed} , \ldots ,GR_{mL}^{renewed} \}$$

When $M$ formations cooperate to execute $N$ tasks in the task list, based on the description of the task network as a tuple, and on the premise that the task type $T_{{{\text{type}}}}$, task space $T_{{{\text{space}}}}$, task index $T_{{{\text{index}}}}$ and task relationship $R$ are known, it is necessary to consider the mapping between task characteristics and formation attributes in order to achieve a reasonable allocation of formations. Thus, the subject $T_{{{\text{force}}}}$ and time $T_{{{\text{time}}}}$ of the task in the task list are determined, and the task network $W = < T,R >$ is constructed.

### Construction of mathematical model

#### Analysis of impact factors

Multiple groups cooperative task allocation adheres to the principle of maximizing the overall operational effectiveness of formations. It optimizes the allocation results of multiple groups for a series of tasks, ensuring that formations can collaborate and efficiently complete the task list. This paper assesses the effectiveness of task allocation from three aspects: task success rate, resource satisfaction, and time satisfaction.

1. Task success rate
2. The task success rate serves as a crucial indicator for evaluating the effectiveness of task allocation, as it directly mirrors the quality and efficiency of task completion. The allocation of formation resources in terms of types and quantities determines the combat power index of the formations. The allocation results of formations are directly associated with combat effectiveness, which in turn determines the success of the task. Taking the task success rate into account in the objective function can guarantee the practicality and effectiveness of the allocation scheme, thereby enhancing the overall reliability of task execution.
3. Resource satisfaction
4. Resource satisfaction examines the relationship between the resource demands of tasks and the resource capabilities of formations, reflecting the fairness and rationality of resource allocation. A proper task allocation should ensure optimal resource configuration, avoiding over consumption of certain resources or resource idleness. If task allocation is improper, it may result in some resources being over utilized while others are left idle, thus affecting the overall execution efficiency. Incorporating the resource satisfaction degree into the objective function contributes to optimizing resource allocation and improving resource utilization efficiency.
5. Time satisfaction
6. Time satisfaction focuses on the time span required for the completion of the task network. A high time satisfaction degree indicates that the task allocation plan can consider the urgency of tasks, arrange the start and end times of tasks rationally, and avoid delays. Meanwhile, it is of great significance for coordinating the temporal relationships between tasks and preventing excessive time conflicts. Incorporating the time satisfaction degree as part of the objective function contributes to improving the timeliness of task execution.

#### Objective function establishment

To enhance the quality and efficiency of task accomplishment and ensure the maximization of resource utilization simultaneously, the constructed objective function should incorporate factors such as task effectiveness, resource utilization rate, and task completion time. Therefore, this paper takes the task success rate, resource satisfaction degree, and time satisfaction degree as the construction factors of the objective function to optimize resource allocation and boost the timeliness of task execution. This achieves the enhancement of both the effectiveness and quality of task allocation.

The objective function of the multi-group collaborative task is determined by the task success rate $F$, resource satisfaction $AGR$ and time satisfaction $PT$, as shown in Eq. (6), and the larger value of the objective function indicates the higher quality of the multi-group task assignment, and on the contrary, the worse quality of the assignment.

6 $$G = F(w_{1} \times AGR + w_{2} \times PT)$$

where $w_{1}$ and $w_{2}$ denote the decision preference weights, which represent the degree of preference of the decision maker for $AGR$ and $PT$, respectively, and satisfy $w_{1} + w_{2} = 1$. When both $AGR$ and $PT$ are more important, in order to avoid tilting the weight values too much to one side and resulting in lower quality of task assignment, $w_{1} ,w_{2} \in \left[ {0.4,0.6} \right]$ is usually used.

1. Task success rate $F$.

The purpose of task success rate is to measure the quality and efficiency of task completion. It not only needs to consider the task combat requirements and the actual combat effectiveness of the formation, but also needs to clarify the impact of upstream tasks on downstream tasks. The success rate of task network depends on the success rate of single task and is related to the importance of single task. The greater the importance and success rate of an individual task, the more significant its impact on the task network. Conversely, lower importance or success rates result in a smaller impact on the network. In addition, the collaboration between upstream and downstream tasks also affects the success rate of task networks. Therefore, this paper measures the success rate of task network from four aspects: task importance, task allocation variable, single task success rate, and cooperative relationship between upstream and downstream tasks.

7 $$\left\{ {\begin{array}{*{20}l} {F = \sum\limits_{j = 1}^{N} {w_{j} } \, \left[ {1 - \prod\limits_{i = 1}^{M} {\left( {1 - u_{ij} p_{ij} } \right)} .\left( {1 - \sum\limits_{h = 1}^{N} {u_{ih} } s_{ijh} } \right)} \right]} \hfill \\ {p_{ij} = C_{{\text{e}}} \frac{{z_{S} }}{{\max (I_{dam} ,z_{S} )}} \times 100\% } \hfill \\ \end{array} } \right.$$

where $w_{j}$ is the importance of the task, indicating the relative importance of the task among all tasks. $u_{ij}$ is the distribution variable of whether the formation $AG_{i}$ executes task $T_{j}$ or not, and takes 1 when $AG_{i}$ executes $T_{j}$; and takes 0 when $AG_{i}$ does not execute $T_{j}$. $p_{ij}$ is the success rate of the formation $AG_{i}$ in executing the task $T_{j}$. $C_{{\text{e}}}$ is the correction coefficient,$0 \le C_{{\text{e}}} \le 1$. $I_{dam}$ is the combat power demand of the task $T_{j}$. $z_{S}$ is the combat power index value of the formation $AG_{i}$, this paper adopts the typical Lanchester equation model to calculate the combat power value. $s_{ijh}$ indicates the synergistic strength of task $T_{h}$ on task $T_{j}$ when the combat force $AG_{i}$ executes task $T_{j}$, and the range of value is $s_{ijh} \in \left[ { - 1,1} \right]$, and the specific value can be determined based on the experience of the experts or the statistical analysis of historical data. Normally, when the logical relationship between task $T_{h}$ and task $T_{j}$ is independent, $s_{ijh}$ takes the value of 0. When the logical relationship between task $T_{h}$ and task $T_{j}$ is facilitated, $s_{ijh}$ takes the value of 1. When the functional relationship between task $T_{h}$ and task $T_{j}$ is dependent, $s_{ijh}$ takes the value of $[0.5,0.8]$. When the functional relationship between task $T_{h}$ and task $T_{j}$ is obstruction type, due to the existence of task $T_{h}$, the combat effectiveness of combat forces in executing task $T_{j}$ is reduced, then $s_{ijh}$ is negative and usually takes the value between $[ - 0.7, - 0.1]$. When the functional relationship between task $T_{h}$ and task $T_{j}$ is facilitative, $s_{ijh}$ is usually positive and takes values in the range of $[0.3,0.5]$. The above value principles are obtained from the experience of a large number of experts and the statistical analysis of historical data, which can be used directly or further adjusted according to the needs of specific scenarios.

(2) Satisfaction with resources $AGR$.

As an important index to measure the relationship between task resource demand and resource ability, resource satisfaction needs to consider the matching degree and redundancy degree of resources. In general, the higher the matching degree of resources and the smaller the redundancy degree, the higher the satisfaction of resources. For a single task, the satisfaction of resources allocated by its grouping can be expressed as Eq. (8).

8 $$AGR_{{T_{d} }} = (\mathop \prod \limits_{{l \in L_{d} }} CR_{{_{l} }}^{d} )^{{{1 \mathord{\left/ {\vphantom {1 {L_{d} }}} \right. \kern-0pt} {L_{d} }}}}$$

where $L_{d}$ is the number of task $T_{d}$ resource requirement types. $CR_{{_{l} }}^{d}$ represents the satisfaction of task $T_{d}$‘s item $l$ resource capability requirements. It is usually determined by the relationship between marshalling resource capability $f_{{sort_{l} }}$ and task resource requirement $CAP_{l}$. When the $f_{{sort_{l} }}$ is too small or too large, the resource matching degree is low or the redundancy degree is high, resulting in lower resource satisfaction.

9 $$CR_{{_{l} }}^{d} = \left\{ {\begin{array}{*{20}l} {\frac{{(1 + 2w_{l} )CAP_{l} }}{{f_{sort} }},f_{{sort_{l} }} > (1 + 2w_{l} )CAP_{l} } \hfill \\ {1,CAP_{l} < f_{{sort_{l} }} \le (1 + 2w_{l} )CAP_{l} } \hfill \\ {\frac{{f_{{sort_{l} }} }}{{CAP_{l} }},f_{{sort_{l} }} \le CAP_{l} } \hfill \\ \end{array} } \right.$$

Then the resource satisfaction of all tasks is defined as Eq. (10).

10 $$AGR = (\mathop {\mathop \prod \limits^{N} }\limits_{(i = 1)} AGR_{i} )^{{{1 \mathord{\left/ {\vphantom {1 N}} \right. \kern-0pt} N}}}$$

(3) Time satisfaction $$PT$$.

Time satisfaction depends on the end time of the task network. The start time and execution time of each task in the task network, and the transfer time between the previous task and the current task will affect the final end time of the task network. The end time should be considered from the task start time and execution time. Therefore, the end time of the task network can be expressed as the sum of the maximum end time and transfer time of the previous task and the duration of the current task.

11 $$st = \max_{{m \in f_{scale} }} \left( {st_{x(m)} + dt_{x(m)} } \right) + I_{{lenT_{n} }}$$

where $x(m)$ represents the $m$th formation in the set of formations $f_{nGroup}$, $st_{x(m)}$ and $dt_{x(m)}$ represent the end time and transfer time of the $m$th formation performing the previous task, respectively. The optimal individual corresponds to the shortest task end time as Eq. (12).

12 $$st_{\min } = \min_{{n = 1,2, \cdots ,N_{size} }} (st(n))$$

Normalizing the execution time and taking the result as time satisfaction $PT$.

13 $$PT(i) = \frac{{st(i) - st_{\min } }}{{\sum\limits_{q = 1}^{{N_{size} }} {(st(} X_{q}^{{}} ) - st_{\min } )}}$$

where $N_{size}$ represents the number of optimization algorithm populations.

#### Determination of constraints

To clarify the boundary constraints and find the optimal or satisfactory solution of the objective function, this paper constructs a multidimensional constraint system under the premise of fully considering the constraints of task allocation, resource capacity, and time and space. Firstly, in the context of task allocation constraints, we put forward a dynamic allocation mechanism grounded in task prioritization to ensure that the sequence and significance of tasks are appropriately manifested. Secondly, with regard to resource capability constraints, we take into account the resource effectiveness assessment to achieve an exact match between resource capability and task requirements. Thereby preventing resource wastage or shortages. Finally, with respect to temporal and spatial constraints, we comprehensively take into consideration time window constraints and spatial accessibility constraints. Through the synergistic computation of time and space dimensions, the comprehensiveness and practicality of the constraints are effectively enhanced, ensuring the high efficiency and feasibility of task execution.

1. Task allocation constraints of formations

In order to improve the satisfaction of resources, it is necessary to analyze the distribution relationship between operational groups and tasks. The allocation relationship between combat group $AG_{i}$ and task $T_{d}$ mainly includes the following two situations: one is that $AG_{i}$ is used for the first time and is directly assigned to deal with task $T_{d}$. The other is that when the same formation undertakes multiple tasks, there is a transfer variable between the tasks, e.g., if $AG_{i}$ is assigned to deal with task $T_{j}$ after dealing with task $T_{d}$, then the transfer variable is set to $f_{ijd} = 1$. Otherwise, $f_{ijd} = 0$. The transfer variable should satisfy the Eq. (14).

14 $$\sum\limits_{j = 1}^{N} {f_{ijd} } \le u_{id}$$

where $u_{id}$ is the allocation variable between task $T_{d}$ and formation $AG_{i}$. During the allocation process, $u_{id}$ is equal to 1 if formation $AG_{i}$ is assigned to task $T_{d}$ and 0 otherwise.

(2) Constraints on task resource requirements

The matching of resource strength and task resource demand is the main factor affecting task completion rate. If a task has specific requirements for resource capabilities, the marshalling forces assigned to that task should also meet those specific requirements. The constraints on task resource capability requirements are shown in Eq. (15).

15 $$CAP_{l}^{d} \le \sum\limits_{m = 1}^{M} {GR_{ml} \times u_{md} }$$

where $CAP_{l}^{d}$ is the capacity demand of task $T_{d}$ on resource $l$.

(3) Spatio-temporal constraints between tasks

The constraints of task execution time and execution area affect task allocation. Therefore, it is necessary to construct corresponding restrictions to avoid the conflict of task execution time, ensure that combat forces arrive at the execution area on time, and improve the effectiveness of task allocation. The execution time of the task needs to satisfy the constraint that the task $T_{d}$ can be executed only after the task $T_{j}$ is completed, in Eq. (16).

16 $${\text{st}}_{d} \ge {\text{st}}_{j} + I_{{len_{j} }} ,j,d = 1,2, \cdots ,N$$

In addition, after completing task $T_{j}$, when proceeding to task $T_{d}$, the formations handling task $T_{d}$ needs to arrive at the execution area. Therefore, the start time of task $T_{d}$ must be no earlier than the time required for the formation $AG_{i}$ to arrive at the region of $T_{d}$.

17 $$\begin{gathered} {\text{st}}_{d} \ge {\text{st}}_{j} + I_{{len_{j} }} + {\text{dt}}_{ij} \hfill \\ = {\text{st}}_{j} + I_{{len_{j} }} + \frac{{d_{jd} }}{{v_{i} }},j,d = 1,2, \cdots ,N \hfill \\ \end{gathered}$$

where $d_{jd}$ is the spatial distance between $T_{j}$ and $T_{d}$. $v_{i}$ is the formation movement speed.

## Multi-component tasking modeling

### The algorithmic process

**Fig. 1:**
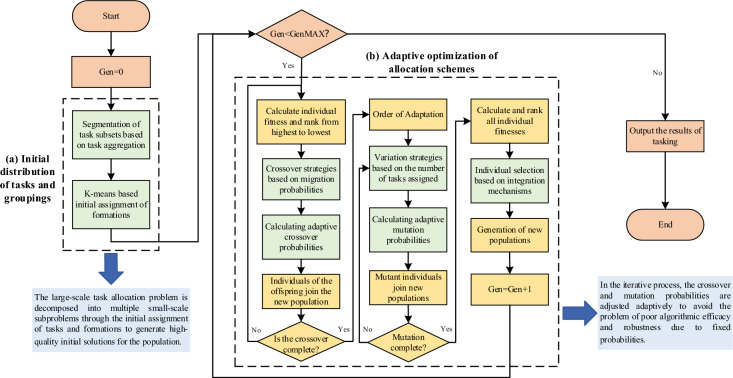
The hierarchical decomposition flowchart of multi-group task network.

Considering the scenarios that multiple groups may collaborate to execute a task, and each formation may execute multiple tasks, this paper adopts the binary matrix method for encoding. For the scenario of M formations executing N tasks, the generated encoding matrix should be of size M × N, whether a formation executes a task is denoted by 0 or 1, the *i*th row describes the set of tasks executed by a formation, and the *j*th column describes the set of formations executing the same task. When M and N are large, the size of the decision space caused is large, which seriously affects the efficiency and quality of the allocation problem solving. In order to improve the algorithm solving efficiency and quality, this paper introduces the K-Means algorithm and adaptive genetic algorithm, and proposes the Hierarchical distribution model based on adaptive genetic algorithm (HDAGA). The algorithm flow chart is shown in Fig. 1:

Description: This figure is divided into two parts: the part of initial assignment of tasks and formations utilizes the K-Means clustering method based on the aggregation degree of tasks to solve the problem of large-scale task network with large-scale decision space and optimization of resource allocation during the initial assignment of large-scale task network. The adaptive optimization part of the task allocation scheme makes use of the dynamic adjustment mechanism in the adaptive genetic algorithm to avoid the problem of local optimal solution in the optimization process of the task allocation scheme and improve the performance of the algorithm.

The main process is divided into:

1. Initial aggregation of tasks and formations based on task aggregation degree

Calculate the task aggregation degree according to the task attribute features and the relationships among tasks to determine the number of task subsets. Then, introduce the K-Means algorithm to analyze the similarity between formations and task subsets in terms of their location and resource requirements. Construct the clustering cost function to achieve the initial allocation of task subsets and formation subsets. This step divides the large-scale task network into multiple smaller subtask networks, which not only effectively reduces the scale of the decision making space for task allocation. Moreover, resource allocation and task scheduling within the clustered task subsets can be optimized in a more targeted manner, thereby laying the foundation for enhancing the solution efficiency of the algorithm.

(2) Adaptive optimization of the task allocation scheme

An adaptive mechanism is introduced into the genetic algorithm (GA). This mechanism dynamically adjusts the intensity and strategies of genetic operations (crossover, mutation, selection). Specifically, it includes a crossover strategy based on migration probability, a mutation strategy based on the number of task allocations, an adaptive crossover and mutation probability calculation method, and a selection strategy based on a fusion mechanism. Subsequently, perform crossover, mutation, and selection operations in sequence to regulate the diversity and convergence of the population, avoiding getting trapped in local optimal solutions. As a result, the algorithm can more flexibly adapt to the changes of the problem, improving the solution quality and efficiency of the algorithm.

### Initial aggregation of tasks and grouping based on task aggregation

**Fig. 2:**
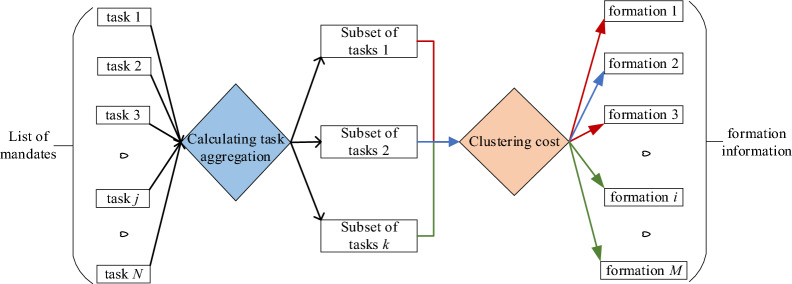
The schematic diagram of the initial allocation of task subsets and formation subsets.

When using genetic algorithms to solve the problem of task allocation with multiple groups, the traditional population initialization method often results in poor chromosome adaptation in the first generation. This inadequacy can lead to the generation of individuals that fail to meet the specified constraints, thereby complicating the iterative optimization process of the algorithm. Aiming at this problem, this paper proposes an initial task and formation allocation strategy based on the degree of task aggregation, aimed at generating high-quality initial solutions for the population. The degree of task aggregation is calculated based on the task attribute features and the relationships, and the number of task subsets is determined to provide a basis for the division of formation groups. Based on the K-Means algorithm, the location and resource demand similarity between the formations and the task subsets are analyzed, and the clustering cost function is constructed to realize the initial allocation of the task subsets and the formation subsets. As shown in Fig. 2, *N* tasks are divided into *K* subsets, and *M* formations are divided into *K* subsets, corresponding to the execution of *K* task subsets, which realizes the initial allocation of task subsets and formation subsets, making the planning of the sequence of tasks performed by subsequent formations more targeted and reducing decision complexity.

#### Calculation of task aggregation degree

Task aggregation is method for grouping multiple similar tasks into a single subset. The task aggregation degree is determined by the similarity in spatial distribution of the tasks, the similarity in resource requirements, and the inter-task relationships. These factors serve as the foundation for dividing the tasks into subsets. The equation for calculating the degree of task aggregation is as Eq. (18).

18 $$r_{jd} = \left\{ \begin{gathered} 1,R_{\log ic} = ^{\prime}and^{\prime} \hfill \\ 0,R_{\log ic} = ^{\prime}or^{\prime} \hfill \\ \frac{{CAP_{j} \cdot CAP_{d} }}{{\left\| {CAP_{j} } \right\|\left\| {CAP_{d} } \right\|}}\frac{{\max d - d_{jd} }}{\max d},other \hfill \\ \end{gathered} \right.$$

When the logical relationship $R_{\log ic} = ^{\prime}and^{\prime}$ between task $T_{j}$ and $T_{d}$ means that there is a close relationship between the two tasks, set $r_{jd} = 1$. If the logical relationship between the two tasks is $R_{\log ic} = ^{\prime}or^{\prime}$, then $r_{jd} = 0$. Otherwise, it needs to be calculated based on the similarity of spatial distribution and the similarity of resource requirements, $d_{jd}$ is the distance between $T_{j}$ and $T_{d}$, and $\max d$ is the farthest distance between tasks in the task list. When the aggregation affiliation between tasks is greater than the threshold value, i.e., $r_{jd} > r_{{th_{1} }}$, that is, the tasks belong to the same subset and the average aggregation degree of the task subset should be less than the threshold value $r_{jd} < r_{th2}$. Otherwise, it is necessary to regroup the task.

#### Formation and initial assignment of tasks

The K-Means algorithm is utilized for the initial allocation of task subsets and formation subsets through the following steps:

*Step 1* Initialize the task subset and formation data information. Normalize the position coordinates and resource requirements, and take the normalized data as samples.

*Step 2* Select the feature sample point in each task subset as the initial clustering cluster point center, denoted as $\alpha_{1}^{0} ,\alpha_{2}^{0} , \cdots ,\alpha_{m}^{0}$.

*Step 3* Euclidean distance is selected as the clustering cost function, as shown in Eq. (19).

19 $$D(c,\alpha ) = \min_{\alpha } \min_{c} \sum\limits_{i = 1}^{M} {\left\| {x_{i} - \alpha_{ci} } \right\|^{2} }$$

where $\alpha$ contains the resource allocation and location of each task subset and $x$ characterizes the resource allocation and location of the formations.

*Step 4* Determine the maximum number of iterations as $t_{\max }$. Repeat Step 5 and Step 6 until $D$ converges.

*Step 5* Calculate the distance from the sample point $x_{i}$ to the center $\alpha_{k}$ of each subcluster and assign the sample to the subcluster with the closest distance.

20 $$C_{i}^{t} = \arg \min_{k} \left\| {x_{i} - \alpha_{m}^{t} } \right\|$$

*Step 6* Based on the sample points belonging to similar clusters, calculate the new cluster class center for that cluster.

21 $$\alpha_{m}^{t + 1} = \arg \min_{\alpha } \left\| {x_{i} - \alpha_{m}^{t} } \right\|$$

*Step 7* Obtain the final clustering result, the points contained in each class cluster are the formation subsets needed to perform the task subsets.

Based on the above steps, the initial allocation of the task subset and the formation group subset is realized, and the initial population is randomly generated according to the combination of the task number and the formation group number, which reduces the decision space for task allocation and lays the foundation for improving the efficiency of subsequent task allocation.

### Adaptive optimization of allocation schemes

In the evolutionary process, the offspring individuals come from two parts: the elite individuals retained through selection and new individuals generated through crossover and mutation operations. The selection, crossover, and mutation operators, along with their associated probabilities, play a crucial role in the optimization capabilities and efficiency of evolutionary algorithms. To enhance the optimization of the allocation scheme, this paper proposes the improvement strategies. (1) A crossover strategy based on migration probabilities is designed to enhance the exploration of the search space through a flexible gene combination approach. (2) A mutation strategy that considers the number of task allocations is proposed to help the algorithm in escaping local optimal solutions. (3) Adaptive methods for calculating crossover and mutation probabilities are used to dynamically adjust the intensity of operations during the evolutionary process, thereby improving the search efficiency of the algorithm. (4) The selection strategies based on the fusion mechanism are constructed to improve the population diversity and enhance the ability to explore global optimal solutions. These strategies dynamically adjust the algorithm parameters to balance global and local searches as well as maintain the population diversity through the change of the fitness value, so as to avoid falling into the local optimum and increase the probability of finding the globally optimal solution. The normalized task effectiveness function is used as the fitness function in the genetic optimization algorithm.

22 $$f(i) = \frac{G(i)}{{\sum\limits_{i = 1}^{{N_{size} }} {G(i)} }}$$

where $N_{size}$ is the number of populations in the adaptive genetic algorithm.

#### Migration probability based crossover strategy

The standard crossover operation is to cut two parent individuals at a certain position and exchange their genetic segments to generate offspring, the parent individuals are randomly determined. To improve the search efficiency and flexibility, this paper proposes a crossover strategy based on migration probability. The crossover parents are adaptively determined by the distribution of population individual fitness, which enhances the global search ability and increases the population diversity.

1. Calculation of migration probabilities

In order to maintain population diversity and improve the global search capability of the algorithm, this paper utilizes the Boltzmann model to represent variations in the fitness of individuals within a population and their mobility. It also calculates the probability of migration for individuals based on their fitness levels. Specifically, for an individual whose fitness is in the kth percentile, its move-in rate $\delta_{k}$ and move-out rate $\varepsilon_{k}$ are calculated by Eqs. (23) and (24), respectively:

23 $$\delta_{k} = \frac{\exp (bk)}{{\sum\limits_{i = 1}^{popsize} {\exp (i)} }}$$

24 $$\varepsilon_{k} = \frac{\exp (b(popsize + 1 - k))}{{\sum\limits_{i = 1}^{popsize} {\exp (popsize + 1 - i)} }}$$

where $popsize$ is the population size and $b$ is the move-in/out factor. From Eqs. (23) and (24), it can be seen that when the individuals in the population have a high degree of adaptation, the emigration rate is higher at this time, and the individuals tend to provide the excellent genes to other individuals; on the contrary, when the individuals in the population have a low degree of adaptation, the migration rate is higher, and the individuals are more inclined to get the excellent genes from other individuals.

(2) The crossover strategy

Roulette is used to select two parent individuals for crossover operation based on the move-in and move-out rates of the individuals, respectively. This approach aims to enhance the fitness of the new individuals through crossover operation and promote the effective exploration of the solution space. The specific crossover process is as follows: first, two chromosomes are obtained from the parents, and one row is randomly selected as the crossover point in the coding matrix of the two parent individuals respectively, and then all the data in the two rows are exchanged to obtain two new chromosomes. At the same time, in order to ensure that the new chromosome can meet the constraints, it needs to be judged for feasibility, such as there is a task that has not been assigned to the coding formations or when the allocation results of a coding group do not meet the task requirements, they are considered to be infeasible, and need to be corrected until the new chromosome is a feasible solution.

**Fig. 3:**
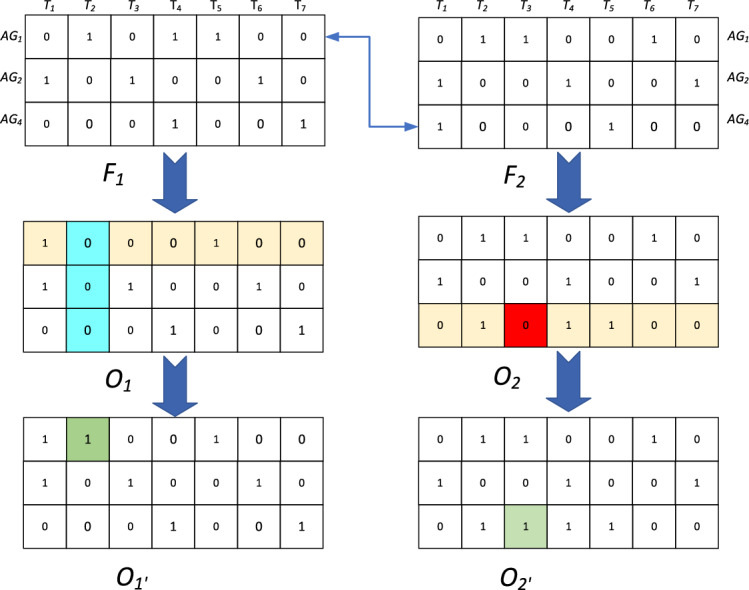
The schematic diagram of the crossover process.

As shown in Fig. 3, new chromosomes *O*_1_ and *O*_2_ are obtained by exchanging row 1 in the paternal *F*_1_ and row 3 in the *F*_2_. It is found that the 2nd task in *O*_*1*_ is not assigned.

to a formation group. It is corrected by adjusting *AG*_1_ to get *O*_1’_, which ensures that each task is executed with a formation group. Relying on *AG*_1_ alone in *O*_2_ cannot satisfy the resource demand of the 3rd task *T*_3_, then *AG*_4_ is adjusted to correct to get *O*_2’_, and then the feasibility is judged.

#### Mutation strategies based on the number of assigned tasks

**Fig. 4:**
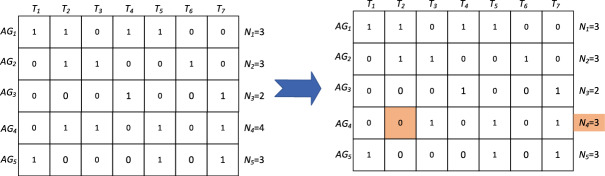
The schematic diagram of the mutation process.

The mutation operation is an important mechanism in genetic algorithms to prevent the population from falling into local minima and to improve the global convergence of the algorithm. The general mutation strategy is to generate a random number to mutate a gene locus on a chromosome, which is more random and not associated with the computation of fitness, which is likely to cause the algorithm to wander blindly in the search space, resulting in slower convergence. When an excessive number of tasks are executed within a specific formation group, it can result in intricate sequential relationships among the tasks. This phenomenon may lead to significant resource redundancy within a formation subset. Additionally, there is a tendency for the overall duration of the task to increase, thereby diminishing the degree of adaptability. Therefore, this paper proposes to sort the tasks in descending order according to the number of tasks executed by the formation groups, and randomly select a task in the set of tasks executed by the first formation group as the part of the chromosome that needs to be mutated, so as to make the mutation strategy more targeted for the enhancement of the adaptation degree. As shown in the Fig. 4, among the allocation results of five formation groups executing seven tasks, $AG_{4}$ needs to execute four tasks, which is the highest number assigned to any formations. After mutating it, $AG_{4}$ executes a total of three tasks of tasks $T_{3} ,T_{5} ,T_{7}$,which is conducive to a more balanced allocation of resources among the formation groups. Similar to the crossover process, it is also necessary to make a feasibility of the new chromosome.

#### Calculation of adaptive crossover and mutation probabilities

In order to avoid the problem that the fixed crossover and mutation probabilities diminishing the effectiveness and robustness of the algorithm, this paper proposes an adaptive method for calculating crossover and mutation probabilities. This approach dynamically adjusts the probabilities of these operations based on both the global fitness of the population and the individual fitness.

1. The strengths $P_{{{\text{g}}c}}$ and $P_{gm}$ of the crossover and mutation operations are determined by the values of the global fitness of the population and are calculated as Eqs. (25) and (26).
  25 $$P_{{{\text{g}}c}} = \left( {P_{c\max } - P_{c\min } } \right)*\cos \left( {\frac{{f_{avg} }}{{f_{\max } }}} \right) + P_{c\min }$$
  26 $$P_{gm} = (P_{m\max } - P_{m\min } )*\left( {1 - \cos \left( {\frac{{f_{avg} }}{{f_{\max } }}} \right)} \right) + P_{m\min }$$
  where $P_{c\max }$ and $P_{m\max }$ are the maximum probabilities of the crossover and the mutation, respectively.$P_{c\min }$ and $P_{m\min }$ are the minimum probabilities of the crossover and the mutation. $f_{\max }$ is the maximum fitness of the chromosomes in the population, and $f_{avg}$ is the average fitness of the chromosomes in the population.

From Eqs. (25) and (26), it can be observed that when the difference in fitness within the population is small, the intensity of the crossover operation is low, while the intensity of the mutation operation is high. At this point, the algorithm exhibits a tendency to converge, which can enhance its convergence speed. Conversely, when the difference in fitness within the population is large, increasing the intensity of the crossover operation can improve the algorithm’s global search capability.

(2) Individual fitness is utilized to guide the adjustment of crossover and mutation probabilities. To prevent radical changes in parameters, the adaptive crossover probability and mutation probability are calculated using the Sigmoid function as Eqs. (27) and (28).
  27 $$P_{c} = \frac{{P_{gc} - P_{c\min } }}{{1 + e^{{10\left( {\frac{{f{\prime} - f_{avg} }}{{f_{\max } - f_{avg} }}} \right)}} }}$$
  28 $$P_{m} = \frac{{P_{gm} - P_{m\min } }}{{1 + e^{{10\left( {\frac{{f{\prime} - f_{avg} }}{{f_{\max } - f_{avg} }}} \right)}} }}$$
  where $P_{c}$ and $P_{m}$ are the population adaptive crossover probability and the population adaptive mutation probability, respectively.$f^{\prime}$ is the fitness function value of the current chromosome. When the fitness value of an individual is smaller, the corresponding crossover and mutation selection probabilities are higher, thus enhancing the optimization ability of the algorithm.

The adaptive adjustment of the operator probability not only combines the global operational intensity but also considers the fitness of the individuals within the population, thereby balancing the exploration and development of the algorithm.

#### Selection strategies based on integration mechanisms

In the selection operation, based on the rule of survival of the fittest, the excellent individuals are retained, while those not adapted to the environment are eliminated. During the selection process, the value of the individual fitness function is employed to measure the adaptability of individuals to the environment. The tournament strategy and the elite retention strategy, as typical selection strategies in genetic algorithms, possess distinct advantages in guiding the optimization process of the algorithm. The tournament strategy involves randomly selecting a certain number of individuals from the population and choosing the individual with the highest fitness as the parent. This operation is repeated until the size of the new population reaches that of the original population. This strategy can not only ensure the elimination of the least fit individuals but also enable the selection of those with relatively lower fitness, safeguarding the diversity of the population and reducing the likelihood of premature convergence. The elite retention strategy dictates that once the population is formed, the chromosomes in the population are sorted according to their fitness values, and the current optimal individuals (or a portion of the optimal individuals) are preserved and directly passed on to the next generation without undergoing crossover and mutation operations. Conversely, individuals with lower fitness generate new individuals through operations such as crossover and mutation. This strategy ensures that the outstanding individuals are not lost due to random factors, thereby maintaining the quality of the solution and accelerating the convergence of the algorithm.

Based on the above analysis, this paper proposes a combination of the elite retention strategy and the two selection strategies of the tournament for chromosome selection. The specific selection strategy is as follows: when selecting parental chromosomes, the chromosomes in the population are sorted according to their fitness function values. A part of the parental chromosomes are selected from the top 30% of the individuals with higher fitness values in the population by means of the elite retention strategy, while the remaining parental chromosomes are selected from the bottom 50% of the individuals with lower fitness values through the tournament strategy. This approach can not only preserve the superior genes of the elite chromosomes but also maintain the diversity of individuals, thus preventing the population from being trapped in a local optimum.

Based on the analysis in Sects. “Initial aggregation of tasks and grouping based on task aggregation” and “Adaptive optimization of allocation schemes”, the pseudo-code for solving the multi-organization task allocation model is shown below.

**Algorithm 1:**
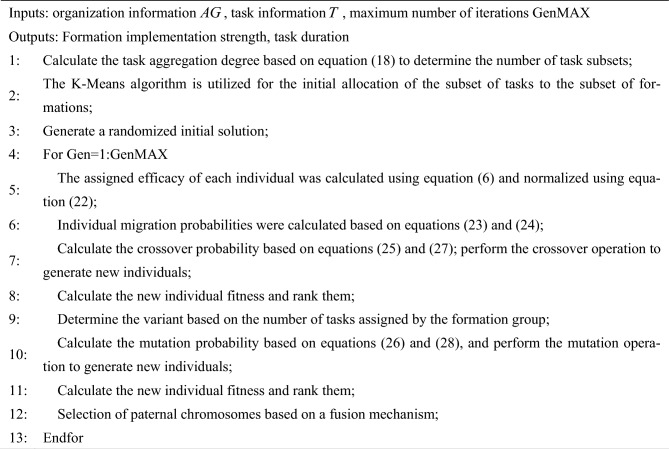
HDAGA-based multi-organization task allocation modeling.

## Experiment and analysis

In this section, Adaptive Genetic Algorithm (AGA), Hybrid Genetic Algorithm (HGA) and Hybrid Discrete Genetic Algorithm (HDGA) algorithms are compared with the proposed algorithm for experiments to verify the effectiveness and universality of this paper’s algorithm.

### Case analysis

#### Method validation and analysis of results

The joint landing task allocation in a scenario is taken as an example to simulate and validate the proposed method. The task list contains 20 tasks, the number of formations is 24, the task space $(X,Y)$ is the location coordinates where the execution tasks are located, different tasks have their own processing time $I_{len}$ in the execution process, and $CAP$ is the resource vector required by the tasks. The attributes of the tasks are detailed in Table 1, while the attributes of formations are outlined in Table 2.

**Table 1:** Attributes of tasks.

Number $p_{id}$	Task name $p_{name}$	Task space (*X*, *Y*)/ (km, km)	Processing time $I_{len}$ /(min)	Resource requirements *CAP*						Significance $w_{j}$
				$l_{1}$	$l_{2}$	$l_{3}$	$l_{4}$	$l_{5}$	$l_{6}$	
1	South beach fire support	(25, 23)	15	0	10	2	2	4	3	0.4
2	North beach fire support	(50, 85)	10	0	8	2	2	3	4	0.3
3	Clearance of mines in sector south	(8, 12)	10	2	0	4	6	0	0	0.4
4	Clearance of mines in sector north	(20, 57)	10	2	0	4	5	0	0	0.4
5	Landing on south beach	(6, 30)	10	2	1	5	5	2	7	0.6
6	Landing on north beach	(41, 72)	10	3	1	4	4	2	8	0.5
7	Sector south defense	(36, 36)	15	3	0	7	10	0	0	0.5
8	Sector north defense	(55, 100)	10	3	1	7	12	0	0	0.5
9	Elimination of blocking forces on the southern road	(120, 20)	10	5	2	7	15	3	1	0.6
10	Elimination of south road mines	(102, 12)	10	1	0	3	5	0	1	0.4
11	Elimination of blocking forces on the northern road	(111, 69)	5	5	1	8	12	3	2	0.5
12	Elimination of north road mines	(115, 89)	5	1	0	3	5	0	1	0.4
13	Bridgehead obstruction	(144, 31)	10	3	1	3	6	1	1	0.5
14	Construct a bridge	(129, 36)	5	0	0	1	3	1	1	0.6
15	Seize the high ground	(84, 42)	15	10	3	12	20	8	5	0.8
16	Highland defense	(109, 51)	15	2	5	6	10	4	1	0.6
17	Destruction of missile launchers	(105, 64)	10	2	0	10	10	0	1	0.8
18	Airport defense	(129, 86)	15	0	2	3	5	4	0	0.4
19	Destruction of missile positions	(133, 57)	20	2	5	4	15	2	1	0.7
20	Port defense	(125, 10)	15	1	1	7	8	4	2	0.4

**Table 2:** Attributes of formations.

Number	Moving speed $v_{m}$/(km/min)	Resource capacity values						Initial position $(X,Y)$/(km, km)
		$l_{1}$	$l_{2}$	$l_{3}$	$l_{4}$	$l_{5}$	$l_{6}$	
AG_1_	2	1	6	2	3	0	1	(60, 60)
AG_2_	2	1	6	2	3	0	1	(40, 70)
AG_3_	2	1	6	2	3	0	1	(18, 25)
AG_4_	2	1	6	2	3	0	1	(20, 20)
AG_5_	2	1	6	2	3	0	1	(6, 38)
AG_6_	4	2	4	5	5	3	4	(50, 20)
AG_7_	4	2	4	5	5	3	4	(5, 82)
AG_8_	4	2	4	5	5	3	4	(38, 58)
AG_9_	4	2	4	5	5	3	4	(25, 15)
AG_10_	4.5	0	3	1	1	6	6	(102, 6)
AG_11_	4.5	0	3	1	1	6	6	(80, 90)
AG_12_	4.5	0	3	1	1	6	6	(108, 77)
AG_13_	4.5	0	3	1	1	6	6	(131, 44)
AG_14_	2.5	2	1	7	7	0	2	(136, 29)
AG_15_	2.5	2	1	7	7	0	2	(42, 84)
AG_16_	3.5	2	1	6	6	0	2	(101, 19)
AG_17_	3.5	2	1	6	6	0	2	(106, 54)
AG_18_	2.75	2	0	8	2	2	4	(126, 69)
AG_19_	2.75	2	0	8	2	2	4	(102, 40)
AG_20_	2.5	2	0	8	2	2	4	(129, 18)
AG_21_	2.5	8	3	1	1	0	5	(30, 92)
AG_22_	3	8	3	1	1	0	5	(125, 39)
AG_23_	3.25	8	3	1	1	0	5	(60, 18)
AG_24_	3.25	8	3	1	1	0	5	(70, 40)

**Fig. 5:**
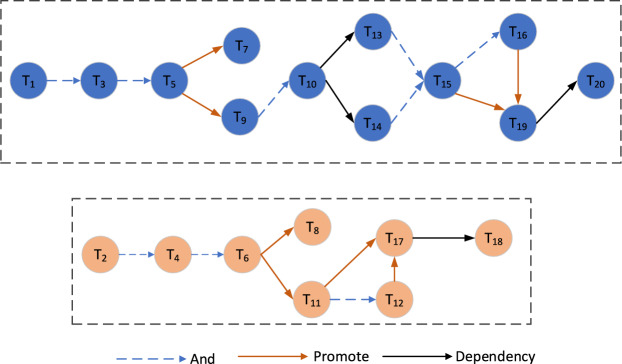
The schematic diagram of relationship between tasks. Describes the relationship between adjacent tasks in two main task branches.

The schematic diagram of inter-task relationships is shown in Fig. 5. The circles in the figure represent tasks, the dashed arrows represent logical relationships, the solid arrows represent functional relationships, and the logical relationships between tasks of different colors are independent of each other.

1. Initial allocation of tasks in multiple groups

Based on the information provided above, the degree of aggregation between tasks is calculated as shown in Table 3.

**Table 3:** The aggregation degree between tasks.

	**1**	**2**	**3**	**4**	**5**	**6**	**7**	**8**	**9**	**10**	**11**	**12**	**13**	**14**	**15**	**16**	**17**	**18**	**19**	**20**
**1**	–	0	**1**	0	**0.74**	0	**0.68**	0	0.33	0.39	0	0	0.22	0.28	0.52	0.45	0	0	0.28	0.33
**2**	0	–	0	**1**	0	**0.80**	0	**0.72**	0	0	0.51	0.47	0	0	0	0	0.49	0.48	0	0
**3**	**1**	0	–	0	**1**	0	**0.81**	0	0.42	0.52	0	0	0.29	0.33	0.56	0.41	0	0	0.29	0.38
**4**	0	**1**	0	–	0	**1**	0	**0.71**	0	0	0.52	0.48	0	0	0	0	0.56	0.35	0	0
**5**	**0.74**	0	**0.82**	0	–	0	**0.75**	0	0.34	0.44	0	0	0.23	0.32	0.55	0.39	0	0	0.24	0.34
**6**	0	**0.80**	0	**1**	0	–	0	**0.71**	0	0	0.56	0.52	0	0	0	0	0.56	0.41	0	0
**7**	**0.68**	0	**0.81**	0	**0.75**	0	–	0	0.55	0.64	0	0	0.44	0.48	**0.73**	0.58	0	0	0.46	0.50
**8**	0	**0.72**	0	**0.71**	0	**0.71**	0	–	0	0	0.66	0.69	0	0	0	0	0.68	0.56	0	0
**9**	0.33	0	0.42	0	0.34	0	0.55	0	–	**0.89**	0	0	**0.86**	**0.88**	**0.78**	**0.82**	0	0	**0.79**	**0.92**
**10**	0.38	0	0.52	0	0.44	0	0.64	0	**0.89**	–	0	0	**0.75**	**0.79**	**0.80**	**0.76**	0	0	**0.69**	**0.86**
**11**	0	0.51	0	0.52	0	0.56	0	0.66	0	0	–	**1**	0	0	0	0	**0.94**	**0.83**	0	0
**12**	0	0.47	0	0.48	0	0.52	0	0.69	0	0	**1**	–	0.00	0	0	0	**0.85**	**0.86**	0	0
**13**	0.21	0	0.29	0	0.23	0	0.44	0	**0.86**	**0.75**	0	0	–	**0.88**	**1**	**0.77**	0	0	**0.83**	**0.83**
**14**	0.28	0	0.33	0	0.32	0	0.48	0	**0.88**	**0.79**	0	0	**0.88**	–	**1**	**0.84**	0	0	**0.86**	**0.84**
**15**	0.52	0	0.56	0	0.55	0	**0.73**	0	**0.78**	**0.80**	0	0	**1**	**1**	–	**1**	0	0	**0.71**	**0.72**
**16**	0.44	0	0.41	0	0.39	0	0.58	0	**0.82**	**0.76**	0	0	**0.77**	**0.84**	**1**	–	0	0	**0.86**	**0.76**
**17**	0	0.49	0	0.56	0	0.56	0	0.68	0	0	**0.94**	**0.85**	0	0	0	0	–	**0.76**	0	0
**18**	0	0.48	0	0.35	0	0.41	0	0.56	0	0	**0.83**	**0.86**	0	0	0	0	**0.76**	–	0	0
**19**	0.28	0	0.29	0	0.24	0	0.46	0	**0.79**	**0.69**	0	0	**0.83**	**0.86**	**0.71**	**0.86**	0	0	–	**0.72**
**20**	0.32	0	0.38	0	0.34	0	0.50	0	**0.92**	**0.86**	0	0	**0.83**	**0.84**	**0.72**	**0.76**	0	0	**0.72**	/

The bold values indicate that the aggregation degree between tasks is relatively high.

**Fig. 6:**
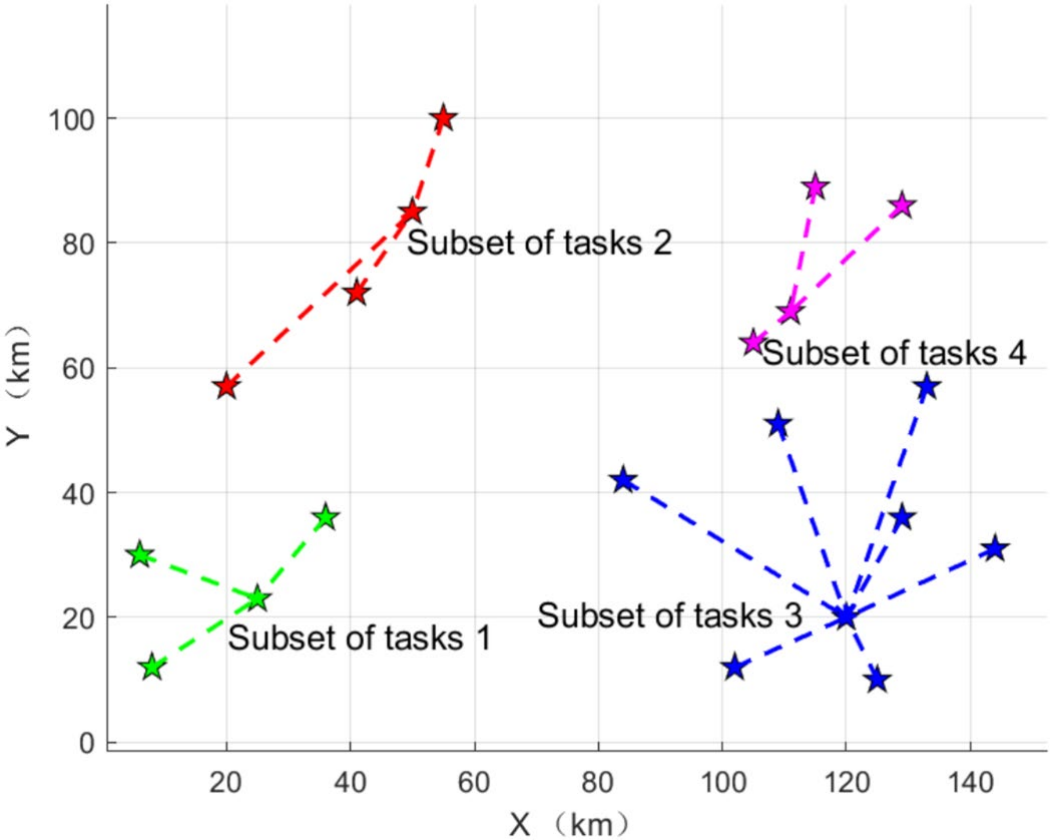
Segmentation results of task subsets. The 20 tasks were divided into 4 groups according to the degree of aggregation among the tasks.

Based on the statistics of inter-task aggregation, the task list is divided into four groups, task subset 1 contains {*T*_1_, *T*_3_, *T*_5_, *T*_7_}, task subset 2 contains {*T*_2_, *T*_4_, *T*_6_, *T*_8_}, task subset 3 contains {*T*_9_, *T*_10_, *T*_13_, *T*_14_, *T*_15_, *T*_16_, *T*_19_, *T*_20_}, and task subset 4 contains {*T*_11_, *T*_12_, *T*_17_, *T*_18_}, as shown in Fig. 6. The average aggregation degrees of the four subsets are 0.80, 0.82, 0.87, and 0.82, respectively. Additionally, the tasks that are independent of each other are classified into different sets, which conforms to the independent relationships between tasks in Fig. 5. The corresponding task locations in different task subsets are more evenly distributed, thus contributing to the overall task execution efficiency. As can be seen from Table 3, although the aggregation between task *T*_7_ and task *T*_15_ is high, if they are divided into the same subset, the average aggregation of subset {*T*_1_, *T*_3_, *T*_5_, *T*_7_, *T*_15_} is 0.71. Similarly, the aggregation of {*T*_7_, *T*_9_, *T*_10_, *T*_13_, *T*_14_, *T*_15_, *T*_16_, *T*_19_, *T*_20_} is 0.76. In this case, the average aggregation of these task subsets is 11.25% and 12.64% lower than that of task subset 1 and task subset 3, respectively.

**Fig. 7:**
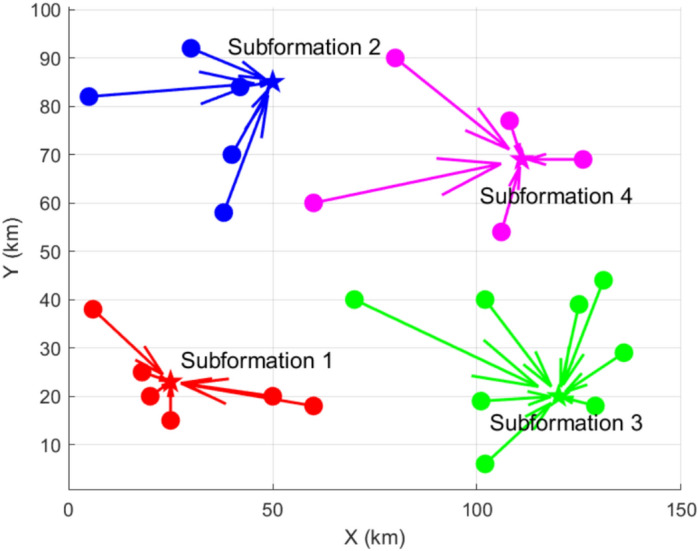
Results of the initial allocation of the task subsets and the formation subsets.The initial assignment of formation subsets and task subsets is realized based on K-Means algorithm.

Based on the K-Means algorithm, the initial allocation of the formation subset and task subset is realized, and the allocation results are shown in Fig. 7, the formation set {*AG*_*3*_, *AG*_4_, *AG*_5_, *AG*_6,_ *AG*_9_, *AG*_23_} executes the task subset 1, the formation set {*AG*_2_, *AG*_7_, *AG*_8_, *AG*_15_, *AG*_21_} executes the task subset 2, and the formation set {*AG*_10_, *AG*_13_, *AG*_14_, *AG*_16_, *AG*_19_, *AG*_20_, *AG*_22_, *AG*_24_} performs task subset 3, and the formation sets {*AG*_1_, *AG*_11_, *AG*_12_, *AG*_17_, *AG*_18_} performs task subset 4. The number of tasks in task subset 3 is high relative to task subset 1, and the number of formations sets assigned is high for high resource demand.

(2) Adaptive optimization of multiple groups task allocation scheme

Based on the above results of the initial allocation of tasks and grouping, the HDAGA algorithm is used for adaptive optimization, and the experimental parameters are set as shown in Table 4.

**Table 4:** Complexity of AGA, HDGA and HDAGA algorithms.

Parameter name	Value
$popsize$	30
$Maxiter$	1000
$X$	4
$P_{c\max }$	0.6
$P_{c\min }$	0.1
$P_{m\max }$	0.2
$P_{m\min }$	0.05
$b$	0.5

**Fig. 8:**
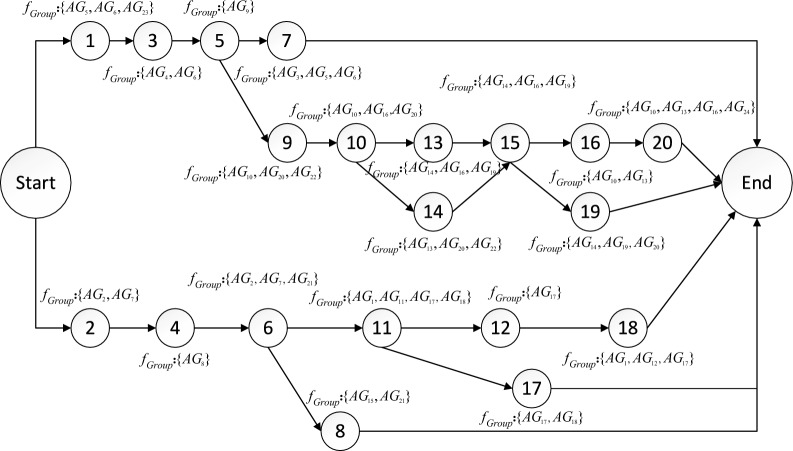
Task network diagram based on HDAGA.

According to the initial task allocation results in Fig. 8, the optimization process of task allocation results based on HDAGA algorithm is carried out to determine the execution power of each task and the order in which each formation executes the tasks. In this test example we focus more on the resource satisfaction AGR and therefore take $w_{1} = 0.6$ and $w_{2} = 0.4$.

Construct a task network diagram, as shown in Fig. 8. The serial number in the circle indicates the number of the task, $f_{Group}$ indicates the serial number of the formations group that executes the current task. The task network diagram flow represents the execution sequence of tasks between task subsets and in task subsets. More precisely, the start time of task subset 3 is the end time of task 5 in task subset 1, and the start time of task subset 4 is the end time of task 6 in task subset 2. Combined with the task details in Table 1, the tasks started with two separate tasks (*T*_*1*_: South Beach Fire Support, *T*_*2*_: North Beach Fire Support) and all subsequent tasks are executed around these two branches, specifically the South Sector Task and North Sector Task. There are six end-of-task conditions, namely the completion of *T*_*7*_, *T*_*19*_, *T*_*20*_, *T*_*18*_, *T*_*17*_ and* T*_*8*_. Each formation is assigned tasks, with one of the formations performing up to four tasks, and each formation performed an average of 2.1 tasks, which allowed the formations to be more evenly distributed among the different tasks overall, with a high rate of resource utilization.

#### Ablation experiment

In order to comprehensively evaluate the effectiveness of this paper’s method, the following is a comparison with the adaptive genetic algorithm (AGA) and hybrid discrete genetic algorithm (HDGA) method to verify the advantages of HDAGA in the allocation of multi-grouping tasks. The basic components of the AGA, HDGA and HDAGA methods are shown in Table 5. The AGA method is to add an adaptive mechanism on the basis of genetic algorithms to realize the HDGA method is based on task clustering and uses genetic algorithm for task reallocation. The HDAGA method in this paper uses genetic algorithm to realize adaptive optimal allocation of multi-group tasks on the basis of the performance advantages of task clustering and adaptive mechanism.

**Table 5:** Comparison of basic building blocks of AGA, HDGA and HDAGA algorithms.

Experimental methods	K-means clustering	Adaptive mechanisms	Genetic algorithm
AGA	×	√	√
HDGA	√	×	√
HDAGA	√	√	√

1. Analysis of task network structure

**Fig. 9:**
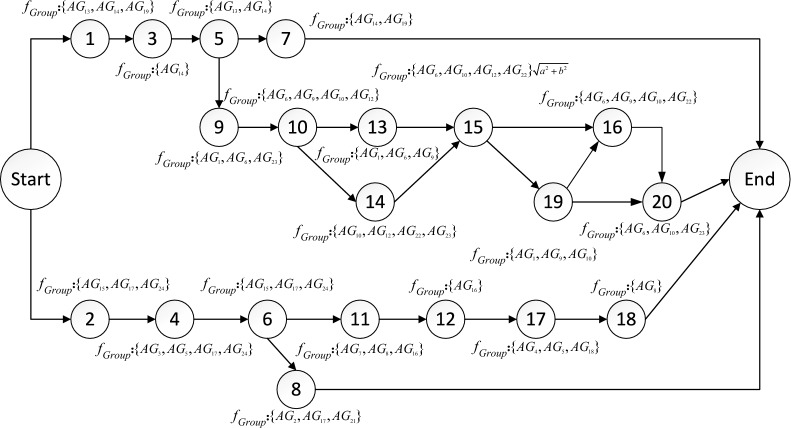
The task network diagram based on AGA.

**Fig. 10:**
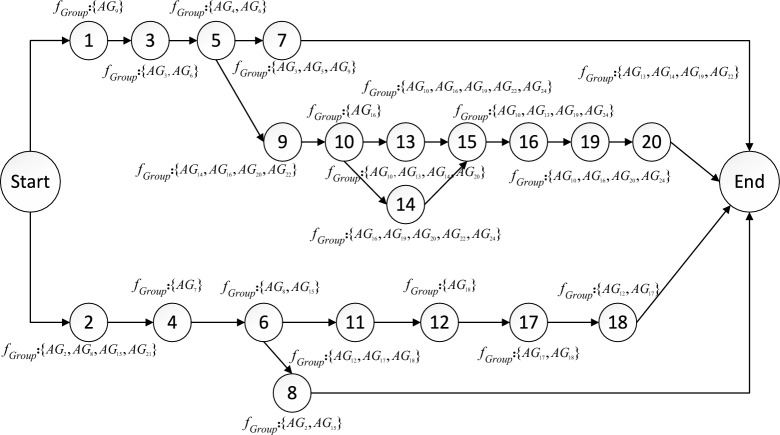
The task network diagram based on HDGA.

Under the constraints of the established logical relationships between tasks, the optimal task network structure obtained by the two algorithms is illustrated in Fig. 9. The task network depicted in Fig. 8 is similar to those of the task networks shown in Figs. 9 and 10, which are also divided into two completely independent branches. The structure of the task network primarily arises from the sequence and the differences in the formation synergies of the tasks, which result in changes to the relationships between them.

For example, in Fig. 8, *AG*_*10*_ and *AG*_*13*_ need to complete task *T*_*16*_ before they can collaborate with other formations to execute task *T*_*20*_, thus constructing the sequential relationship between *T*_*16*_ and *T*_*20*_. In Fig. 9, *AG*_*6*_ needs to complete task *T*_*13*_. *AG*_*10*_, *AG*_*12*_ and *AG*_*22*_ need to complete task *T*_*14*_, creating the sequential relationship between *T*_*15*_ and both *T*_*13*_ and *T*_*14*_. In Fig. 10, *AG*_*24*_ is required to perform tasks *T*_*15*_, *T*_*16*_, and *T*_*19*_, resulting in a sequential relationship among the three tasks.

The longest task sequence in the task network obtained based on HDAGA is 9, while the longest task sequence in the task network obtained based on AGA and HDGA is 10. The end conditions of the task network based on AGA and HDGA are the completion of *T*_*7*_, *T*_*20*_, *T*_*18*_, and *T*_*8*_. In contrast, the number of tasks executed in parallel as determined by HDAGA is greater, which reduces the closeness of the correlation between tasks.

In summary, compared to the task networks obtained based on AGA and HDGA, each task in the network generated by HDAGA can be scheduled and executed more independently. This independence enhances both the efficiency and flexibility of task execution.

In Fig. 9, it can be seen that formation groups *AG*_*6*_ and *AG*_*10*_ are both assigned six tasks, formation groups *AG*_*9*_, *AG*_*14*_ and *AG*_*17*_ are assigned four tasks, and formation groups *AG*_*11*_ and *AG*_*20*_ are not assigned any tasks, leading to an uneven allocation of tasks among the formation groups. In Fig. 10, it can be seen that formation groups *AG*_*1*_, *AG*_*11*_, and *AG*_*23*_ are also not assigned tasks, resulting in resource redundancy. These findings indicate that the task allocation based on AGA and HDGA for multiple groups groups leads to resource wastage.

(2) Analysis of task execution efficiency and algorithm complexity

Task execution efficiency and algorithm complexity are the key indexes of ablation experiments, in which task execution efficiency is used to assess the effectiveness of the algorithm, mainly considering the task execution time, resource consumption, etc. When the task execution time is shorter and the resource utilization rate is higher, it indicates that the task execution efficiency is higher, i.e., the effectiveness of the algorithm is higher.

Based on the AGA, HDGA, and HDAGA task networks to analyze the timing relationship among tasks, the sequential relationships of each task are analyzed. Table 6 presents the start and end times for task execution across the different task networks, with the red numbers indicating the end times corresponding to each branch task within the networks.

**Table 6:** Implementation time of the task network.

ID	AGA		HDGA		HDAGA	
	Start time/min	End time/min	Start time/min	End time/min	Start time/min	End time/min
1	44.465	59.465	6.294	21.294	12.103	27.103
2	18.288	28.288	14.396	24.396	11.275	21.275
3	67.564	77.564	26.356	36.356	32.165	42.165
4	44.703	54.703	34.396	44.396	21.275	31.275
5	84.808	94.808	36.356	46.356	42.165	52.165
6	65.026	75.026	44.396	54.396	31.275	41.275
7	107.046	122.046	46.356	61.356	52.165	67.165
8	83.506	93.506	66.269	76.269	53.148	63.148
9	94.808	104.808	56.356	66.356	56.356	62.165
10	109.732	119.732	78.156	88.156	78.156	80.044
11	83.506	93.506	54.396	59.396	41.275	46.275
12	85.804	90.804	66.750	71.750	52.053	57.053
13	131.256	141.256	98.156	108.156	93.215	103.215
14	119.732	124.732	105.481	110.481	108.965	113.965
15	156.698	171.698	128.989	143.989	127.615	142.615
16	178.341	193.341	153.651	168.651	153.243	168.243
17	90.804	100.804	80.906	90.906	64.247	74.247
18	100.804	115.804	100.207	115.207	83.549	98.549
19	177.195	197.195	182.265	202.265	178.138	198.138
20	237.352	252.352	202.265	217.265	178.023	193.023

As shown in Table 6, the end times for the six different branches (*T*_*7*_, *T*_*19*_, *T*_*20*_, *T*_*18*_, *T*_*17*_, *T*_*8*_) in Fig. 8 are 67.165, 198.138, 193.023, 98.549, 74.247, and 63.148, respectively. The maximum end time, which represents the point at which the entire list of tasks is completed, is 198.138. In the case of the same list of tasks, the AGA-based and HDGA-based algorithms calculate the end times as 252.352 and 217.265, respectively. Comparing the end time of each branch, the end time of *T*_*7*_ based on HDGA is earlier than the end time of *T*_*7*_ based on HDAGA. However, comparing the three task networks, it can be seen that the task *T*_*7*_ is a separate branch at the end of the task *T*_*5*_, and the duration of the task is shorter, resulting in a lesser impact on overall task execution efficiency. The end times of other branches calculated based on HDAGA are shorter than those derived from AGA and HDGA. In summary, compared to AGA and HDGA, the proposed method enhances the execution efficiency of multiple groups allocation results by 23.5% and 11.2%, respectively.

In order to test the stability of the algorithms, the three algorithms run independently for ten times within the scenario. The average execution time and the shortest execution time of the task network are counted, as shown in Table 7. The average time and the shortest time of the task execution of the allocation scheme obtained based on the proposed method are shorter, and the efficiency of task execution is higher. Compared with AGA and HDGA, the average execution time is improved by 22.6% and 12.0%.

**Table 7:** Implementation time of the task network.

Methods	Average time/min	Minimum time/min
AGA	250.728	239.2
HDGA	220.45	209.664
HDAGA	194.076	189.493

The complexity of an algorithm is mainly used to measure the computational resources (e.g., time, space, etc.) required by the algorithm under different input sizes, and its available Big *O* expression to evaluate the growth rate of the algorithm in the worst case scenario provides a way to compare the complexity among different algorithms. In the paper, assuming that the population size of the genetic algorithm is *N*, the number of iterations *T*, and the latitude of the optimization sequence is *D*, the complexity comparison of the AGA, HDGA and HDAGA algorithms is shown in Table 8.

**Table 8:** Complexity of AGA, HDGA and HDAGA algorithms.

Methods	Initialization complexity	Fitness function	Iterative Complexity of Chromosome Updates	K-means clustering complexity	Total complexity
AGA	$$O\left( {N*D} \right)$$	$$O\left( {N*T} \right)$$	$$O\left( {T*N*D} \right)$$	–	$$O\left( {T*N*D} \right)$$
HDGA	$$O\left( {N*D} \right)$$	$$O\left( {N*T} \right)$$	$$O\left( {T*N*D} \right)$$	$$O\left( {K*N*D*I} \right)$$	$$O\left( {T*N*D} \right)$$
HDAGA	$$O\left( {N*D} \right)$$	$$O\left( {N*T} \right)$$	$$O\left( {T*N*D} \right)$$	$$O\left( {K*N*D*I} \right)$$	$$O\left( {T*N*D} \right)$$

As shown in Tables 7 and 8, the HDAGA algorithm performs optimally in terms of task execution time, and its execution efficiency is significantly higher than that of the AGA and HDGA algorithms. Meanwhile, HDAGA is consistent with AGA and HDGA in terms of algorithmic complexity and does not add additional computational burden due to the performance improvement.

(3) Analysis of convergence

**Fig. 11:**
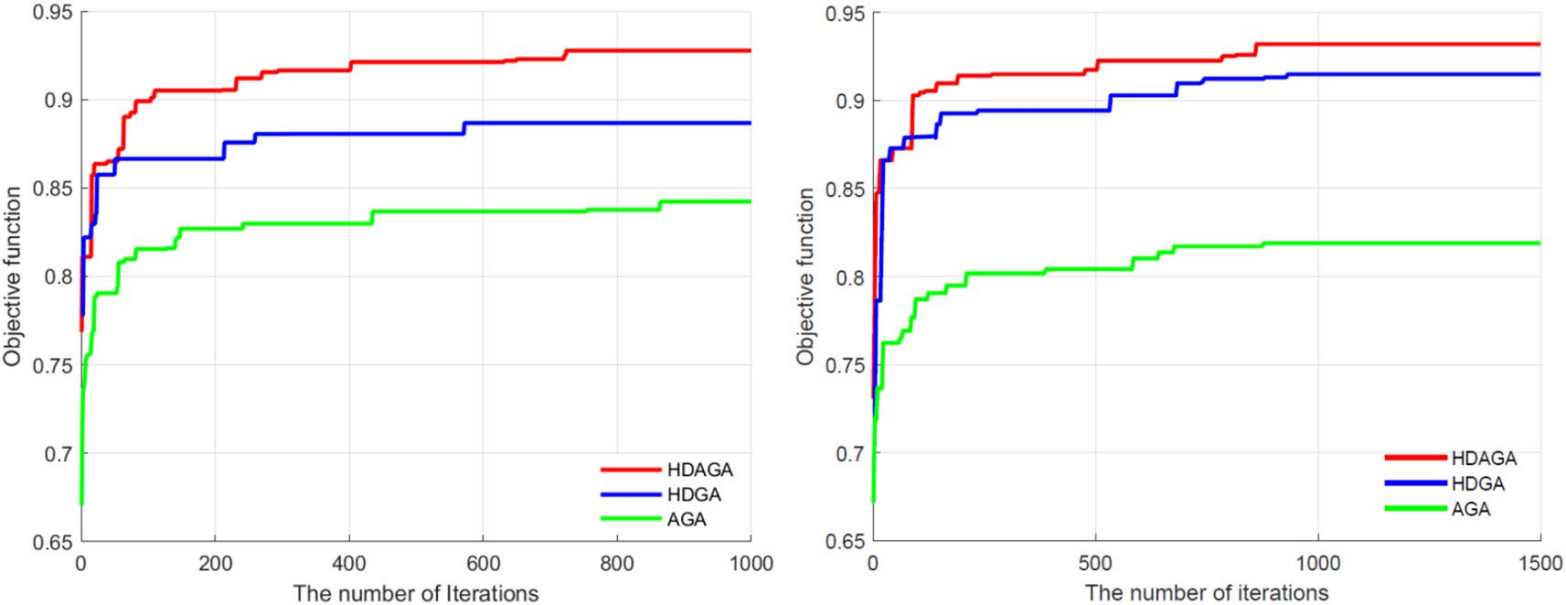
Convergence curve of the objective function (**a**) 1000 iterations, (**b**) 1500 iterations.

Under the same parameters, the convergence change curves of the objective function of different algorithms are compared, as shown in Fig. 11a. Based on the HDAGA algorithm in the first 110 iterations of the objective function to improve faster, showed a better global search ability, and in the number of iterations from 110 to 230 iterations can jump out of the local optimal solution, and ultimately in 290 iterations after the gradual convergence. Compared with the HDAGA algorithm in this paper, although the HDGA algorithm also shows a better ability to search for an optimal solution in the initial stage of iteration, it falls into the local optimal solution after 50 iterations, and jumps out of the local optimal solution after 200 iterations, but there is not a large improvement, and the final convergence value is 0.887, which is lower than the convergence value of the objective function obtained by this paper’s algorithm, which shows that the introduction of the adaptive mechanism improves the algorithm’s the global search capability of the algorithm. As can be seen in Fig. 11a, the initial values of HDGA and HDAGA are 0.778 and 0.768, respectively, which are much larger than the initial value of 0.670 obtained by the AGA. In addition, the objective function value of the AGA converges to 0.842 in 860 iterations. This result shows that the introduction of clustering algorithms can improve the quality and efficiency of the task assignment, and verifies that the proposed adaptive genetic algorithm has a better searching ability than the traditional genetic algorithm. traditional genetic algorithm with better optimization finding ability.

In addition, we also plotted Fig. 11b with 1500 iterations as the iteration termination condition. Comparing Fig. 11a and Fig. 11b, it can be seen that the objective function values of the HDAGA algorithm converge to 0.926 and 0.932, respectively, and the difference between the two is very small, which indicates that the results of the method in this paper have good stability.

### Test set analysis

To further illustrate the advantages of the proposed method, considering the differences in the number of formations and the number of tasks, 10 test sets with different problem sizes are constructed, and the proposed method is compared with AGA, HDGA, and HGA, respectively. In order to enhance the reliability of the experimental results, 20 independent experiments are conducted for each test set. The average convergence time and task allocation objective function values of different algorithms in the test sets are calculated as metrics to assess the reliability and stability of the optimization capabilities of the algorithm. The comparison results are shown in Table 9.

**Table 9:** Comparative analysis of results in test sets.

Test sets		Convergence time/s				Value of the objective function/*f*			
		AGA	HDGA	HGA	HDAGA	AGA	HDGA	HGA	HDAGA
1	N = 7; M = 15	4.631	2.875	3.086	2.853	0.853	0.902	0.923	0.942
2	N = 15; M = 7	5.932	3.859	3.428	3.058	0.688	0.759	0.817	0.853
3	N = 15; M = 15	6.428	4.058	3.765	2.965	0.704	0.849	0.809	0.876
4	N = 15; M = 20	6.672	4.169	4.264	3.073	0.727	0.863	0.874	0.885
5	N = 15; M = 30	7.028	4.729	5.829	3.174	0.662	0.716	0.768	0.790
6	N = 30; M = 15	8.137	4.973	6.117	3.390	0.756	0.794	0.804	0.804
7	N = 30; M = 20	11.364	5.075	7.809	3.206	0.780	0.817	0.806	0.827
8	N = 30; M = 30	13.592	5.426	8.325	3.695	0.73	0.821	0.800	0.845
9	N = 30; M = 40	12.633	5.953	8.641	3.732	0.768	0.779	0.812	0.837
10	N = 60; M = 30	17.269	6.227	10.748	4.463	0.656	0.680	0.705	0.739

Based on the analysis of the results from various test sets, the HDAGA demonstrates the fastest convergence and the highest quality of allocation across in all test sets. With the increase of the problem-solving scale, the convergence time of different algorithms increases, and the corresponding task allocation quality shows a decreasing trend, especially the change trend of the AGA is most obvious. However, the advantages of the HDAGA become more evident as the complexity of the task allocation problem increases. In the ten test sets, the average convergence time is 6.768 s for AGA, HDGA, and HGA, while it is 3.361 s for HDAGA, resulting in an average improvement of 3.407 s in convergence time. The average objective function value is 0.7807 for AGA, HDGA, and HGA, compared to 0.8398 for HDAGA. The average objective function value for task allocation shows an improvement of 7.58%.

**Fig. 12:**
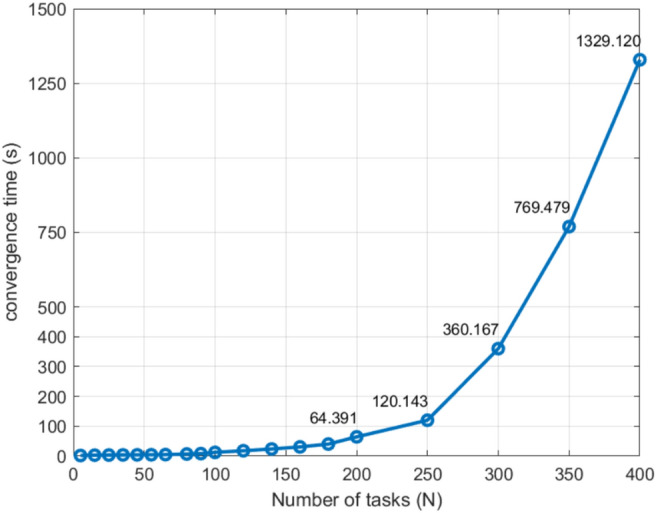
Convergence time plot.

In order to measure the operation of this paper’s algorithm under different sizes of tasks, this paper tested the model under the amount of 1–400 tasks and plotted the convergence time curve, as shown in Fig. 12.

As can be seen from Fig. 12, when the task size is less than 185, the convergence time of this paper’s method for effective task allocation is less than 60 s. When the task size is 200, the algorithm converges in 64.391 s. When the task size reaches 250, the algorithm converges in 120.143 s. When the task size is larger than 250, the convergence time shows a larger increase with the task size The convergence time is 120.143 s. This shows that the method in this paper has a faster convergence time when dealing with medium and small tasks with a number of scales less than 200, but it can also be applied to deal with large tasks with a number of scales from 200 to 400, and its convergence time is up to 1329.12 s.

## Conclusions

Based on multi-dimensional information encompassing task attribute characteristics, convoluted relationships among tasks, the spatial arrangement of formations, and resource allocation patterns, this paper achieves the hierarchical decomposition of the convoluted relationships among tasks via the partitioning of task subsets, the initial assignment of formation subsets, and the specification of the execution task sequence for each formation. Thereby constructing an intuitive and potent task network to furnish decision-making support for commanders. The distinctiveness of this paper is manifested in the following aspects:

1. This paper defines the task network and meticulously refine the binary group variables within the network. This action furnishes a well-structured and systematic framework for multiple groups collaborative tasks.
2. When compared with the AGA algorithm, the proposed algorithm in this paper improves the average allocation efficiency and average allocation quality by 54.12% and 14.66% on case simulation and multiple test sets, respectively. This result validates that the initial allocation of tasks and formations based on the task aggregation degree can effectively enhance the quality of the initial solution. This provides a firm basis for decreasing the solution space of large-scale task network allocation.
3. By comparing with HDGA and HGA algorithms, the proposed algorithm significantly enhances the allocation efficiency and allocation quality on case simulation and multiple test sets. The average improvement rates are 38.53% and 4.34% respectively. This indicates that the algorithm proposed in this paper can adaptively adjust the operation operators to enhance its ability to find the optimal solution within a complex task network.

In this paper, based on inter-task relationship constraints, the mapping allocation between multiple groups and multiple tasks is realized, which enhances the quality of task allocation. The paper focuses on key constraints such as task success rate, resource satisfaction, and time satisfaction. However, during the execution of a specific task, it may be restricted by the terrain. Consequently, in future research, the incorporation of formation movement cost and additional resource loss as constraints under terrain restricted conditions will be contemplated to enhance the performance of multiple groups task assignment.

## Data Availability

The datasets used and analyzed during the current study are available from the corresponding authors upon reasonable request.

## References

[1] Gao, X. et al. Conditional probability based multi-objective cooperative task assignment for heterogeneous UAVs. *Eng. Appl. Artif. Intell.* **123**, 106404. 10.1016/j.engappai.2023.106404 (2023).

[2] Peng, M. et al. Automatic milp model construction for multi-robot task allocation and scheduling based on large language models. Preprint at 10.48550/arXiv.2503.13813 (2025).

[3] Agrawal, A., Bedi, A.S., Manocha, D. Rtaw: An attention inspired reinforcement learning method for multi-robot task allocation in warehouse environments. In* 2023 IEEE International Conference on Robotics and Automation (ICRA)*. 1393–1399 (2023). 10.1109/ICRA48891.2023.10161310.

[4] Liu, Z. et al. GA-DRL: Graph neural network-augmented deep reinforcement learning for DAG task scheduling over dynamic vehicular clouds. *IEEE Trans. Netw. Serv. Manag.*10.1109/TNSM.2024.3387707 (2024).

[5] Li, T., Wang, G. & Fu, Q. MADDPG-D2: An intelligent dynamic task allocation algorithm based on multi-agent architecture driven by prior Knowledge. *CMES-Comput. Model. Eng. Sci.*10.32604/cmes.2024.052039 (2024).

[6] Ye, F., Chen, J., Sun, Q., Tian, Y. & Jiang, T. Decentralized task allocation for heterogeneous multi-UAV system with task coupling constraints. *J. Supercomput.* **77**, 111–132. 10.1109/ICUAS48674.2020.9213989 (2021).

[7] Wang, J. F., Jia, G. W., Lin, J. C. & Hou, Z. X. Cooperative task allocation for heterogeneous multi-UAV using multi-objective optimization algorithm. *J. Cent. South Univ.* **27**, 432–448. 10.1007/s11771-020-4307-0 (2020).

[8] Wei, T. & Zhong, J. Towards generalized resource allocation on evolutionary multitasking for multi-objective optimization. *IEEE Comput. Intell. Mag.* **16**, 20–37. 10.1109/MCI.2021.3108310 (2021).

[9] Bi, W., Shen, J., Zhou, J. & Zhang, A. Heterogeneous multi-UAV mission reallocation based on improved consensus-based bundle algorithm. *Drones.*10.3390/drones8080345 (2024).

[10] Samiei, A. & Sun, L. Distributed matching-by-clone Hungarian-based algorithm for task allocation of multiagent systems. *IEEE Trans. Rob.* **40**, 854–863. 10.1109/TRO.2023.3335656 (2023).

[11] Liu, Y. et al. A spherical vector-based adaptive evolutionary particle swarm optimization for UAV path planning under threat conditions. *Sci. Rep.* **15**, 2116. 10.1038/s41598-025-85912-4 (2025). 39814793 10.1038/s41598-025-85912-4 PMC11735934

[12] Saadaoui, H., El, B. F. & Illi, E. Information sharing based on local PSO for UAVs cooperative search of moved targets. *IEEE Access* **9**, 134998–135011. 10.1109/COMMNET.2018.8360276 (2021).

[13] Zhou, X. & Yang, K. Cooperative multi-task assignment modeling of UAV based on particle swarm optimization. *Intell. Decis. Technol.* **18**, 919–934. 10.3233/IDT-230760 (2024).

[14] Wang, S. et al. Cooperative task allocation for multi-robot systems based on multi-objective ant colony system. *IEEE Access* **10**, 56375–56387. 10.1109/ACCESS.2022.3165198 (2022).

[15] Senthil, K. A. M. & Venkatesan, M. Multi-objective task scheduling using hybrid genetic-ant colony optimization algorithm in cloud environment. *Wirel. Pers. Commun.* **107**, 1835–1848. 10.1007/s11277-019-06360-8 (2019).

[16] Garcia, C. E., Camana, M. R. & Koo, I. ACO-based scheme in edge learning NOMA networks for task-oriented communications. *IEEE Access.*10.1109/ACCESS.2024.3374635 (2024).

[17] Katoch, S., Chauhan, S. S. & Kumar, V. A review on genetic algorithm: Past, present, and future. *Multimed. Tools Appl.* **80**, 8091–8126. 10.1007/s11042-020-10139-6 (2021). 33162782 10.1007/s11042-020-10139-6 PMC7599983

[18] Ye, F., Chen, J., Tian, Y. & Jiang, T. Cooperative task assignment of a heterogeneous multi-UAV system using an adaptive genetic algorithm. *Electronics* **9**, 687. 10.3390/electronics9040687 (2020).

[19] Ye, X., Guo, H. & Luo, Z. Two-stage task allocation for multiple construction robots using an improved genetic algorithm. *Autom. Constr.* **168**, 105583. 10.1016/j.autcon.2024.105583 (2024).

[20] Bayrak, A. E. & Polat, F. Employment of an evolutionary heuristic to solve the target allocation problem efficiently. *Inf. Sci.* **222**, 675–695. 10.1016/j.ins.2012.07.050 (2013).

[21] Mishra, M. et al. Context-aware decision support for anti-submarine warfare mission planning within a dynamic environment. *IEEE Trans. Syst. Man Cybern. Syst.* **50**, 318–335. 10.1109/TSMC.2017.2731957 (2017).

[22] Ruan, C., Zhou, Z., Liu, H. & Yang, H. Task assignment under constraint of timing sequential for cooperative air combat. *J. Syst. Eng Electron.* **27**, 836–844. 10.21629/JSEE.2016.04.12 (2016).

[23] Kumar, H. & Tyagi, I. Hybrid model for tasks scheduling in distributed real time system. *J. Ambient. Intell. Humaniz. Comput.* **18**, 2881–2903. 10.1007/s12652-020-02445-6 (2021).

[24] Wu, M. et al. Evaluation of particle swarm optimization, genetic algorithms, and ant colony optimization in autonomous mobile robots scheduling: A comparative study. In *2024 IEEE International Conference on Industrial Technology (ICIT)* 1–5 (IEEE, 2024). 10.1109/ICIT58233.2024.10540680.

[25] Wu, Z. A comparative study of solving traveling salesman problem with genetic algorithm, ant colony algorithm, and particle swarm optimization. In *Proceedings of the 2020 2nd International Conference on Robotics Systems and Vehicle Technology* 95–99 (2020). 10.1145/3450292.3450308.

[26] Han, S., Xiao, L. An improved adaptive genetic algorithm. In *SHS web of conferences. EDP Sciences* vol. 140 01044. 10.1051/shsconf/202214001044 (2022).

[27] Zheng, Q., Yue, C., Zhang, S., Yao, C. & Zhang, Q. Optimal allocation of water resources in canal systems based on the improved grey wolf algorithm. *Sustainability* **16**, 3635. 10.3390/su16093635 (2024).

[28] Li, Y. B., Sang, H. B., Xiong, X. & Li, Y. R. An improved adaptive genetic algorithm for two-dimensional rectangular packing problem. *Appl. Sci.* **11**, 413. 10.3390/app11010413 (2021).

[29] Xie, J., Li, X., Gao, L. & Gui, L. A hybrid genetic tabu search algorithm for distributed flexible job shop scheduling problems. *J. Manuf. Syst.* **71**, 82–94. 10.1016/j.jmsy.2023.09.002 (2023).

[30] Bao, Q., He, C. J., Sui, Y., Hou, M. & Zhang, G. X. Troops demanding analysis based on exponent-Lancheser model. *Armament. Autom.* **27**, 19–29 (2008).

[31] Park, H. S., Kwon, Y. H., Seo, J. H. & Woo, B. H. Distributed hybrid genetic algorithms for structural optimization on a PC cluster. *J. Struct. Eng.* **132**, 1890–1897. 10.1061/(ASCE)0733-9445(2006)132:12(1890) (2006).

[32] Kucukkoc, I., Aydin, K. G., Karaoglan, A. D. & Karadag, S. A hybrid discrete differential evolution–genetic algorithm approach with a new batch formation mechanism for parallel batch scheduling considering batch delivery. *Int. J. Prod. Res.* **62**, 460–482. 10.1080/00207543.2023.2233626 (2024).

[33] Han, Y., Yan, X. & Gu, X. Novel hybrid discrete differential evolution algorithm for the multi-stage multi-purpose batch plant scheduling problem. *Appl. Soft Comput.* **115**, 108262. 10.1016/j.asoc.2021.108262 (2022).

[34] Yang, H. J. & Lee, H. S. Evidence-based smart home service process for lighting energy saving. *J. Archit. Inst. Korea Plan. Des.* **31**, 15–25. 10.5659/JAIK_PD.2015.31.8.15 (2015).

[35] Wang, J. & Bao, X. Research on the international communication of Chinese culture—A case study of the “Chinese bridge” online programs. *Open J. Soc. Sci.* **13**, 202–218. 10.4236/jss.2025.132014 (2025).

[36] Gao, W., Luo, J., Zhang, W., Yuan, W. & Liao, Z. Commanding cooperative ugv-uav with nested vehicle routing for emergency resource delivery. *IEEE Access.* **8**, 215691–215704. 10.1109/ACCESS.2020.3040790 (2020).

